# A naturally occurring canine model of syndromic congenital microphthalmia

**DOI:** 10.1093/g3journal/jkae067

**Published:** 2024-04-29

**Authors:** Leonardo Murgiano, Esha Banjeree, Cynthia O'Connor, Keiko Miyadera, Petra Werner, Jessica K Niggel, Gustavo D Aguirre, Margret L Casal

**Affiliations:** Department of Clinical Sciences & Advanced Medicine, University of Pennsylvania, Philadelphia, PA 19104, USA; Sylvia M. Van Sloun Laboratory for Canine Genomic Analysis, University of Pennsylvania, Philadelphia, PA 19104, USA; Department of Pathobiology, School of Veterinary Medicine, University of Pennsylvania, Philadelphia, PA 19104, USA; Section of Medical Genetics, School of Veterinary Medicine, University of Pennsylvania, Philadelphia, PA 19104, USA; East Bridgewater Veterinary Hospitla, East Bridgewater, MA 02333, USA; Department of Clinical Sciences & Advanced Medicine, University of Pennsylvania, Philadelphia, PA 19104, USA; Section of Medical Genetics, School of Veterinary Medicine, University of Pennsylvania, Philadelphia, PA 19104, USA; Genetic Diagnostic Laboratory, Perelman School of Medicine, University of Pennsylvania, Philadelphia, PA 19104, USA; Department of Clinical Sciences & Advanced Medicine, University of Pennsylvania, Philadelphia, PA 19104, USA; Sylvia M. Van Sloun Laboratory for Canine Genomic Analysis, University of Pennsylvania, Philadelphia, PA 19104, USA; Department of Clinical Sciences & Advanced Medicine, University of Pennsylvania, Philadelphia, PA 19104, USA; Sylvia M. Van Sloun Laboratory for Canine Genomic Analysis, University of Pennsylvania, Philadelphia, PA 19104, USA; Department of Clinical Sciences & Advanced Medicine, University of Pennsylvania, Philadelphia, PA 19104, USA; Section of Medical Genetics, School of Veterinary Medicine, University of Pennsylvania, Philadelphia, PA 19104, USA

**Keywords:** microphthalmia, syndrome, canine, anemia, thrombocytopenia, *DNAJC1*

## Abstract

In humans, the prevalence of congenital microphthalmia is estimated to be 0.2–3.0 for every 10,000 individuals, with nonocular involvement reported in ∼80% of cases. Inherited eye diseases have been widely and descriptively characterized in dogs, and canine models of ocular diseases have played an essential role in unraveling the pathophysiology and development of new therapies. A naturally occurring canine model of a syndromic disorder characterized by microphthalmia was discovered in the Portuguese water dog. As nonocular findings included tooth enamel malformations, stunted growth, anemia, and thrombocytopenia, we hence termed this disorder Canine Congenital Microphthalmos with Hematopoietic Defects. Genome-wide association study and homozygosity mapping detected a 2 Mb candidate region on canine chromosome 4. Whole-genome sequencing and mapping against the Canfam4 reference revealed a Short interspersed element insertion in exon 2 of the *DNAJC1* gene (g.74,274,883ins[T^70^]TGCTGCTTGGATT). Subsequent real-time PCR-based mass genotyping of a larger Portuguese water dog population found that the homozygous mutant genotype was perfectly associated with the Canine Congenital Microphthalmos with Hematopoietic Defects phenotype. Biallelic variants in *DNAJC21* are mostly found to be associated with bone marrow failure syndrome type 3, with a phenotype that has a certain degree of overlap with Fanconi anemia, dyskeratosis congenita, Shwachman–Diamond syndrome, Diamond–Blackfan anemia, and reports of individuals showing thrombocytopenia, microdontia, and microphthalmia. We, therefore, propose Canine Congenital Microphthalmos with Hematopoietic Defects as a naturally occurring model for *DNAJC21*-associated syndromes.

## Introduction

Eye development is relatively comparable between mammals (Gelatt 2020); after gastrulation, the neuroectoderm develops forming the neural groove, which folds on each side and fuses into the neural tube, the precursor of central nervous system (Hyttel 2010; Saraiva and Delgado 2020). There, an optic pit forms on each side of the forebrain region, developing into an optic vesicle invaginating into the optic cup, while the optic stalk progresses to form the optic embryonic fissure. All these embryonic tissues will originate the known ocular structures (Hyttel 2010).

Ocular maldevelopment is responsible for approximately 30% of global vision impairment and blindness in humans. Still, due to the wide phenotypic spectrum, including multisystemic features, along with unknown or complex etiology and unclear pathogenesis, these congenital disorders pose a major diagnostic challenge, with treatment options limited to symptomatic or supportive care. The prevalence of congenital microphthalmia is estimated to be 0.2–3.0 per 10,000 individuals, with nonocular involvement reported in ∼80% of cases (Llorente-Gonzalez *et al.* 2011; Searle *et al.* 2018). In humans, at least 30 types of syndromic microphthalmia have been described, with at least 40 associated genetic loci identified; nonocular manifestations include nose and facial dysmorphia, dental malformations, agenesis of the brain, heart defects, mental retardation, and digital abnormalities, among others (Verma and Fitzpatrick 2007).

Inherited eye diseases are widely and descriptively characterized in the dog, and investigation of canine ocular disease models have played an important role in the identification and understanding of genes relevant for ocular development and function. Furthermore, canine models have also proven to be an excellent platform for the development of new therapies for retinal diseases (Acland *et al.* 2001; Komáromy *et al.* 2010; Beltran *et al.* 2012; Mellersh 2014; Guziewicz *et al.* 2018; Miyadera *et al.* 2022). Microphthalmia is a common congenital ocular malformation in dog and has been described to occur both sporadically and in a familial manner in multiple canine breeds including Akita, Australian Shepherd, Beagle, Bedlington Terrier, Cavalier King Charles Spaniel, Collie, Dachshund, Doberman Pinscher, Great Dane, Miniature Schnauzer, Old English Sheepdog, Poodle, Saint Bernard, and Soft-Coated Wheaten Terrier, and very often in mixed breed dogs (Gelatt 2014; Saraiva and Delgado 2020). Microphthalmia is usually identified in young dogs with a median age of diagnosis of 7.5 months, with no statistically significant sex predisposition. Kaukonen and colleagues observed one form of microphthalmia in dogs less than 10 weeks of age and characterized its maternal inheritance mechanism (Kaukonen *et al.* 2018); Shaw and colleagues detected microphthalmia in dogs ranging in age from 2 months to 5 years. (Shaw *et al.* 2019; Gelatt 2020). Genetic variants in *RAB3GAP1* have been shown to cause a polyneuropathy comparable to Warburg syndrome in the Alaskan Husky, Rottweiler, and Black Russian terrier breed, with manifestations including microphthalmia (Wiedmer *et al.* 2015; Mhlanga-Mutangadura, Johnson, Ashwini *et al.* 2016; Mhlanga-Mutangadura, Johnson, Schnabel *et al.* 2016). Microphthalmic eyes have various degrees of vision impairment (Slatter 1990). Very often, nictitating membrane protrusion and entropion due to the lack of eyelid support is observed (Gould and McLellan 2014).

The phenotypic variability of microphthalmia can be classified into three main groups: (1) isolated microphthalmia, (2) microphthalmia combined with other ocular defects (cataracts, microphakia, retinal dysplasia or folds, glaucoma, ocular dermoids, and others), and (3) a whole multiorgan condition, including nonocular manifestations consistent with syndromic microphthalmia (Saraiva and Delgado 2020).

In dogs, nonocular manifestations are varied. Ocular manifestations can be found associated with deafness and albinism described in miniature schnauzers (Gelatt and Gum 1981; Zhang *et al.* 1991) alongside neuronal vacuolation-associated polyneuropathy in boxers (Geiger *et al.* 2009). Microphthalmia, congenital cataracts, posterior lenticonus, and retinal dysplasia have been observed in Akita (Laratta *et al.* 1985) and severe bilateral microphthalmos in a Pomeranian (Dell 2010). Microphthalmia has also been described in a Doberman Pinscher along with dysgenesis, hyperplastic primary vitreous, and retinal dysplasia (Peiffer and Fischer 1983). In Portuguese water dogs (PWDs), microphthalmia with multiple anterior segment defects has been reported (Shaw *et al.* 2019). The merle gene is a color dilution gene that lightens the coat color; the most severe ocular anomalies occur in homozygous merles with abundant white hair coat involving the head region. The ocular abnormalities that occur with this condition include microphthalmia (Hyttel 2010).

The PWD breed population has gained popularity and grown in numbers. Nonetheless, the breed originates from three major founding events involving two different kennels. Such limited genetic diversity initially prompted genetic studies of GM1 gangliosidosis and thus the identification of the canine ß-galactosidase gene and its genetic variant (Saunders *et al.* 1988; Wang and Wiggs 2014) followed shortly thereafter by identification of the *PRCD* gene variant as causally associated with a prevalent form of inherited blindness (Goldstein *et al.* 2006; Zangerl *et al.* 2006). More recent studies include Addison's disease in the current breed population, (Chase *et al.* 2005, 2006; Sutter *et al.* 2007) and retinal degeneration caused by a genetic variant in *CCDC66* (Murgiano *et al.* 2020).

Shaw and colleagues characterized histologic findings of microphthalmia in PWDs, describing lens abnormality and often multiple additional anterior segment defects (Shaw *et al.* 2019). Other histologic lesions were present in a plurality of cases, such as the lack of both the ciliary cleft and trabecular meshwork (47%) and abnormal relationship of anterior segment structures (also 47%). Many of the lesions Shaw and colleagues described were similar to those reported in anterior segment dysgenesis in humans (Reis and Semina 2011; Shaw *et al.* 2019). Two PWDs presented other problems: one had lissencephaly and one had chondrodysplasia.

In this paper, we describe the clinical and molecular basis of naturally occurring canine model of a syndromic disorder characterized by microphthalmia as the main ocular phenotype affecting PWDs, presumably different from the one described by Shaw *et al.* (2019). The syndromic manifestation also includes variable nonocular findings, such as stunted growth, anemia, and thrombocytopenia. We termed the syndrome Canine Congenital Microphthalmos with Hematopoietic Defects (cCMHD).

## Methods

### Ethical statement

The research was conducted in full compliance and strict accordance with the Association for Research in Vision and Ophthalmology (ARVO) Resolution on the Use of Animals in Ophthalmic and Vision Research. The protocol was approved by the Institutional Animal Care and Use Committees (IACUCs) of the University of Pennsylvania (#806301, 804197, POAP #513).

### Sample collection and phenotype assessment

A total of 68 privately owned dogs (*n* = 42 affected and *n* = 26 unaffected relatives; age range: 0.5–75 months) were enrolled in the study. A physical examination and, where possible, ophthalmologic assessments were performed using binocular indirect ophthalmoscopy and biomicroscopy after pupillary dilatation; blood samples were collected for complete blood cell counts and serum biochemistry screens and/or processed for molecular studies. In selected cases, close-up images of the eyes were taken with a Genesis-D portable retinal camera set for taking images of anterior segment abnormalities. In two cases and seven unrelated mix-breed control dogs’ axial length of the globes was measured using an Accutome B-Scan ultrasound unit (cases) or a DGH Technology Inc, Model 5000e A-scan ultrasound instrument (normal controls). The cases were examined at 14 weeks of age, and the seven controls ranged from 11.7 to 14 weeks of age.

For each dog, eye examination records and pedigree information were obtained. There was complete ancestry information of all the cases. All the available clinical eye information was reviewed by a board-certified veterinary ophthalmologist (GDA) prior to the inclusion of each animal in the study. All the available bloodwork and clinical information was reviewed by MLC.

### Family tree

Available ancestry information was parsed and plotted using the R package kinship2 (Sinnwell *et al.* 2014) in the R Studio and GenAbel integrated environment (Aulchenko *et al.* 2007). We built two family trees: a representative one in the main body of text encompassing only 23 cases, and a complete one with all the 42 cases, included in the Supplementary material due to its size.

### DNA and RNA extraction from blood

DNA from blood samples, cases, and controls, was extracted using DNAeasy Blood and Tissue Kit by Qiagen following manufacturer's instructions. Additionally, blood from two cases was also collected in a Paxgene vial (Qiagen) and RNA extracted following the manufacturer's instructions. Nucleic acid concentration was assessed by Nanodrop.

### Critical interval mapping

A cohort of 18 PWD cases and 20 PWD controls was genotyped on a 220k Illumina CanineHD BeadChip and used for the analysis. In addition, we merged the dataset with that of 23 not affected by microphthalmia (KM) PWDs previously genotyped on a 170k Illumina CanineHD SNP chip (Murgiano *et al.* 2020). It resulted in 171,159 shared SNPs for the analysis filtered using the command “–extract”. Individuals and SNPs were selected using the commands “–keep”, and “–extract”, while final files were generated through the “–merge” and “–recode” commands.

Genome-wide association study (GWAS) was carried out using the R package GenABEL (Aulchenko *et al.* 2007) in R Studio. During the preliminary step of the analysis, we used the standard quality control settings to remove markers and individuals with call rates <95%, markers with minor allele frequency (MAF) < 5%, and markers strongly deviating from Hardy–Weinberg equilibrium (*P* < 10^−8^). The mixed model analysis followed was carried out as designed in the GenABEL package. For the QQ-plot we used qqman. GWAS analysis was followed by a homozygosity mapping approach, carried out with PLINK v1.9 (Chang *et al.* 2015) to detect extended intervals of homozygosity with shared alleles and fine-map the region containing the responsible mutation. Homozygosity analysis was carried out on all cases and controls using the commands “–dog”, “–homozyg”, and “–homozyg-group”, on the merged dataset. Positions (of SNP data and WGS data, see below) were remapped to UU_Cfam_GSD_1.0 (Canfam4) for SNP data and Canfam3 as part of the filtering pipeline, using the NCBI coordinate remapping service (https://www.ncbi.nlm.nih.gov/genome/tools/remap, accessed 06/22/2023).

### Whole-genome sequencing

An Illumina TruSeq PCR-free DNA library for case MP021 was prepared, with an insert size of 350 bp. The sequenced dog was a case homozygous for the candidate haplotype. After collecting HiSeq2500 paired-end reads (2× 150 bp), the fastq files were created using Casava 1.8. A total of 346,731,103 paired-end reads were collected. The paired-end reads were then mapped to the UU_Cfam_GSD_1.0, Canfam4 canine reference. The reads were aligned using Burrows-Wheeler Aligner (BWA) version 0.5.9-r16 (Li and Durbin 2009) using the default settings. The SAM file generated by BWA was then converted to BAM, and the reads sorted using samtools (Li *et al.* 2009). A total of 346,283,005 reads were mapped. The average coverage for the bam file was 21.46, appropriate for a recessive disease variant discovery pipeline.

### Genomic variants calling

The GATK version 4 program (McKenna *et al.* 2010) was used for variant calling of the aligned data within the candidate interval, using the “HaplotypeCaller” module. The variant data for each sample was obtained in variant call format (vcf, version 4.0), as were raw calls for all samples and sites flagged using the standard variant filtration module. Variant filtration followed the best practice documentation. SnpEff software (Cingolani *et al.* 2012) and the UU_Cfam_GSD_1.0 (Canfam4) assembly were used to predict the functional effects of the variants detected. Large genetic variants were detected through DELLY2 (Rausch *et al.* 2012) and compared to a database of bam file our group generated from internal projects and the 10k canine genome project, and against the DBVDC after coordinate conversion (Jagannathan *et al.* 2019). The variants were filtered against control vcfs using bcftools (Li 2011). Each variant was carefully checked against the annotation.

### Genotyping

The *DNAJC21* insertion was verified in the cases and the available controls by re-sequencing targeted PCR products by Sanger sequencing. For the initial confirmation and sequencing of the variant, PCR primers were designed using PRIMER3 (Untergasser *et al.* 2012). PCR products were amplified using flanking primers for the exon 2 insertion, F (5′-TTTACTTGTTTGCTGACTGAATATCTG-3′, CFA4: 74,274,960–74,274,986) and R (5′-TCTAAGGTTCCTTCTGACCCTAATACT −3′ CFA4: 74,274,686–74,274,712). Amplification was carried out with Invitrogen Platinum SuperFi with the following parameters: The wild-type amplicon spans 301 bp, while the mutant is 384 bp. Sequence data were visualized with 4Peaks (https://nucleobytes.com/4peaks/). For mass genotyping, we employed a real-time PCR protocol with the following primers F (5′-GCTCTCTCCTGAGGGTCACT-3′, CFA4: 74,274,851–74,274,870) and R (5′-GGATAATGCCGCAGAAGCC-3′; CFA4: 74,274,913–74,274,931). The reporter Primers F (5′-AAATTAATCCAAGCAGCATAT-3′, CFA4: 74,274,880–74,274,900) and R (5′-AATCCAAGCAGCAAAA-3′, CFA4: 74,274,884–74,274,937) and the following parameters: 95°C 10′, and 50× cycles of 95°C 15″, 64°C 1′. All the coordinates here reported are Canfam4. PCR products were run on 1.5% agarose gel, 0.5 μg/mL ethidium bromide.

### RNA analysis

PCR products were amplified from cDNA obtained from RNA extracted from blood of two cases, using flanking primers designed to fall within exon 2; F (5′-TTTACTTGTTTGCTGACTGAATATCTG-3′, CFA4: 74,274,960–74,274,986) and R (5′-TCTAAGGTTCCTTCTGACCCTAATACT-3′ CFA4: 74,274,686–74,274,712). This allowed a short wild-type amplicon (68 bp) and a relatively short mutant amplicon of ∼140 bp. PCR was run with the following parameter: 98°C 10′, and 35× cycles of 98°C 10″, 60°C 10″, 72°C 35″. PCR products were run on 2% agarose gel, 0.5 μg/mL ethidium bromide. The mutant product was sequenced by Sanger sequencing.

## Results

**Fig. 1.**
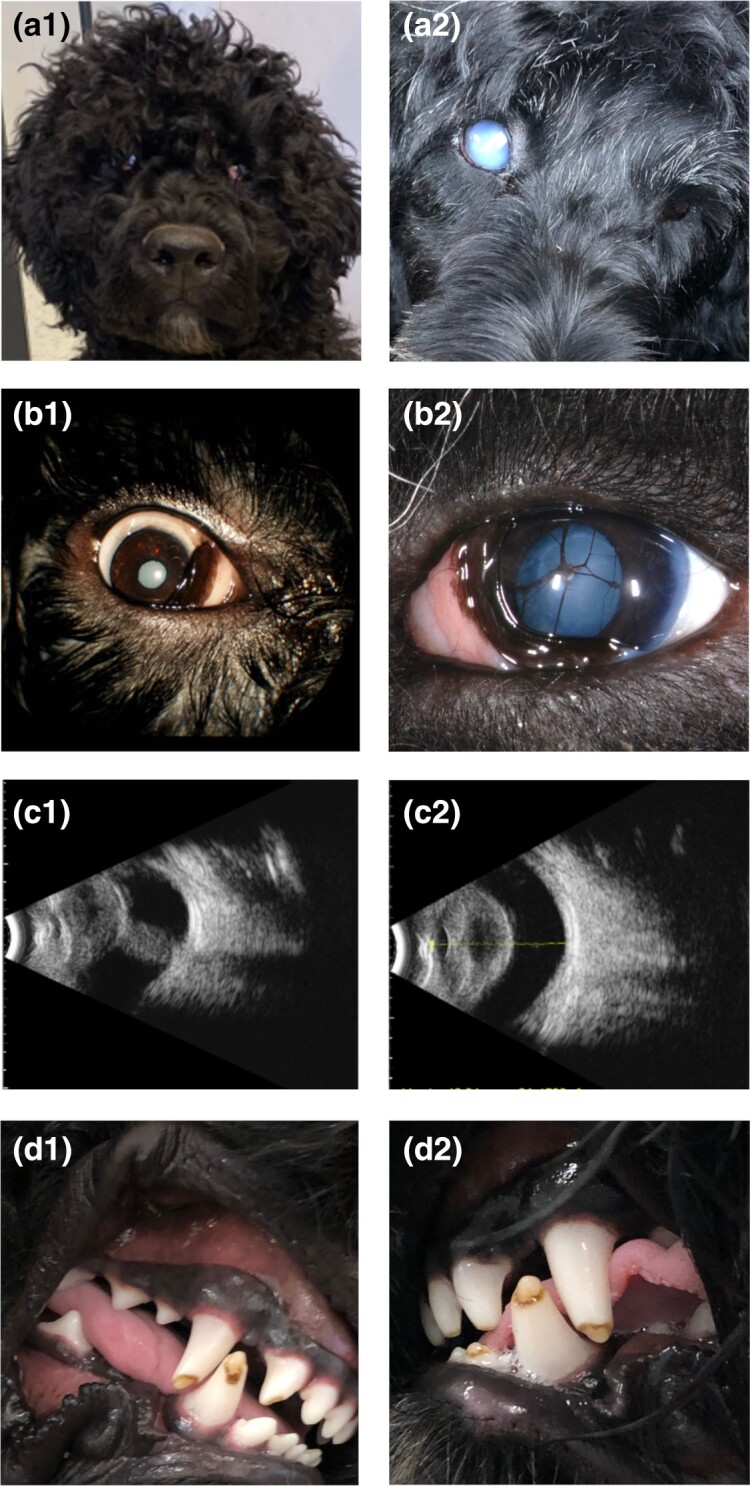
a–c) Ocular manifestations of cCMHD in affected PWDs. a) Facial feature (a1) and detail (a2) of an affected PWD with complete cataract in the right eye and microphthalmia in the left eye. b1) Right eye of bilateral microphthalmia affected dog with complete. Note protrusion of the nictitating membrane, increased scleral show, and decreased corneal diameter. b2) Microphthalmic left eye with complete cataract and persistent pupillary membrane Ci/C2. B-scan ocular ultrasound of an affected PWD (4-months old) with bilateral microphthalmia showing decreased axial length of the globe in each eye (c1 right eye; c2 left eye). The right eye (c1) has a prominent vascular lesion (persistent hyperplastic primary vitreous) extending from the optic disc to the posterior lens capsule d1 and d2: dental malformation in cCMHD, manifesting in enamel hypoplasia and discoloration.

A total of 68 privately owned dogs (42 affected and 26 unaffected relatives; age range: 0.5–75 months) were enrolled in the initial study. Ascertainment of ocular phenotype was made by a board-certified veterinary ophthalmologist (GDA) or by one of the authors (CO'C) who worked with the ACVO diplomate on this project during her pediatrics/genetics residency at the University of Pennsylvania, and subsequently. Bilateral or unilateral microphthalmia (< ∼50% of a normal eye size) was observed in all affected dogs (five dogs were examined because microphthalmia was diagnosed by the referring veterinarian but the word microphthalmia did not appear in the ophthalmology report). Beyond this shared microphthalmia, a subset of dogs also showed defects more generally associated with anterior segment dysgenesis (cataract, corneal dystrophy, microphakia/aphakia, glaucoma, and persistent pupillary membranes) (Fig. 1).

Diagnosis was made based on gross clinical observations with a pen light/Finoff transilluminator-small eye size in relation to the palpebral fissure and orbit, prominence/prolapse of the third eyelid as small globe recessed into the orbit. In two cases, B-scan ultrasounds were done in three eyes of 14-week-old dogs; the fourth eye could not be measured reliably because of the persistent hyperplastic primary vitreous. The axial length of the three globes ranged from 12 to 13 mm. For the seven unrelated controls, 11.7–14 weeks of age, the axial globe length mean ± 1 SD was 19.1 ± 0.97 mm for the right eye, and 19.0 ± 0.66 for the left eye. Beyond this shared microphthalmia, a subset of dogs also showed defects more generally associated with anterior segment dysgenesis (cataract, corneal dystrophy, microphakia/aphakia, glaucoma, and persistent pupillary membranes) (Fig. 1). Some of the microphthalmia-affected dogs also showed retinal dystrophy, detachments, atrophy, or folds (Table 1).

**Table 1.** Ocular changes associated with microphthalmia syndrome in 42 affected PWD (15 males and 27 females).

Ocular changes	# of dogs	Notes
Microphthalmia	37^a^	Bilateral (36); unilateral (1)
Cataracts	29	*Bilateral*: Complete (15); incomplete/immature (7); unspecified (6). *Asymmetric*: Focal R & complete L (1).
Persistent pupillary membranes	20	Iris to iris, bilateral (15) or unilateral (2); unspecified, bilateral (2); iris to iris R & iris to lens L (1)
Glaucoma	8	Unilateral (5); bilateral (3)
Microphakia/aphakia	7	Microphakia, bilateral (2); aphakia, unilateral (3) or bilateral (2)
Retinal lesions	6	Detachment, bilateral (3); atrophy, bilateral (1); fold & atrophy L (1); unspecified, unilateral (1)
Synechia	4	Anterior, unilateral (2); posterior R & anterior L (1); anterior + posterior R & posterior L (1)
Lens resorption	2	Unilateral (1); bilateral (1)
Corneal dystrophy	2	Unilateral (1); bilateral (1)

L, left eye; R, right eye.

^*a*^The remaining 5 dogs were examined for their microphthalmia but the word microphthalmia did not appear in the ophthalmology report.

**Fig. 2.**
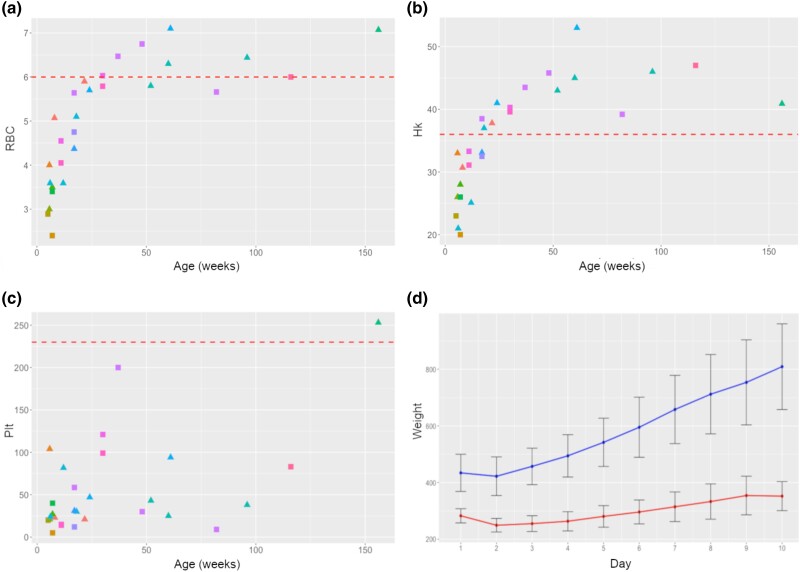
Blood and growth signs of cCMHD. a–c) Weekly (*x*-axis) blood analyses of cases. Males (7) are indicated with squares, females (9) with triangles. Some dogs had blood drawn at multiple time points. For each case blood was taken at one to three different time points. Age is expressed in weeks. The dotted line indicates expected values’ lower threshold. a) Red blood cell (RBC) counts in 10^6^/uL different ages in PWDs with cCMHD (normal range: 5.7–8.5 × 10^6^/uL). b) Hematocrit (Hk) % at different ages (normal range: 41–58%). c) Platelet (Plt) count at 10^3^/uL at different ages (normal range: 186–545 × 10^3^/uL). Interestingly, all the platelets remain consistently under threshold barring two individuals. d) Average and SD of the growth curves of PWD controls (upper) and affected by cCMHD (bottom). Weights are expressed in grams. Four males’ and five females’ weights were recorded. Observe the stunted growth of affected puppies.

**Fig. 3.**
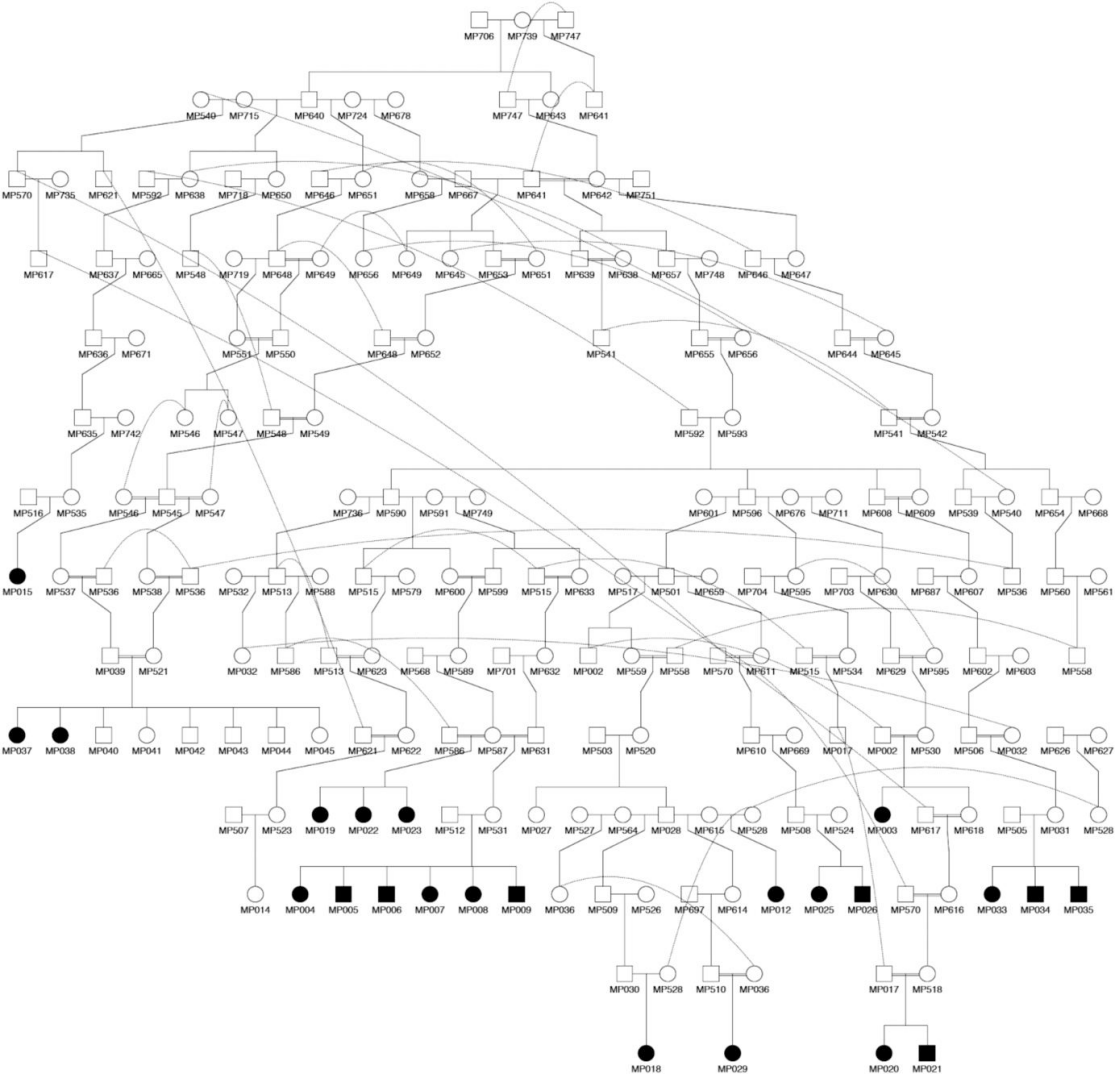
Family tree of a subset of 23 cases whose ancestry information was available. Males are marked in squares, females in circles. Affected dogs are marked in black, clinically normal with empty squares and circles. For clarity, unaffected siblings have been removed from most families—see the MP39-MP521 litter for an example of a typical outcome. Curved lines link multiple representations of a specific individual occurring in more than one spot of the family tree. Complete family tree in Supplementary File 1.

Thrombocytopenia with normocytic normochromic anemia was a very frequent hematologic abnormality found in all the available cases (*n* = 28) in which blood panels were performed. Several large platelet clumps were however detected. The abnormalities noted in most dogs that had blood work performed were decreased red blood cell counts, low hematocrit, and thrombocytopenia (decreased platelet numbers) (Fig. 2). In one of these cases (MP001 in Fig. 3), the platelet counts were low and did not change over time, but the hematocrits were always within normal range for the age. In another case (MP011 in Fig. 3), there was no information on hematocrits, but the platelets were noted to be low at 7 weeks of age and normal at 26 weeks of age. In a third case, the hematocrit dramatically improved but the platelet count improved only moderately over time (MP019). Overall, as the affected dogs get older, their red blood cell counts and hematocrits improved but the platelets generally remained low in all the dogs that were possible to measure (Fig. 2a–c). Interestingly, only one of the dogs with a low platelet count showed delayed clotting after a traumatic injury.

To a lesser extent, the oculo-hematologic phenotype was accompanied by enamel hypoplasia and delayed tooth eruption (Fig. 1d). Puppies that were not euthanized very early in life were reported to be stunted in growth, as shown in their weights plotted over the first 10 days of life in two affected puppies of a litter of nine (Fig. 2d).

Of the 42 cases, we were initially able to gather complete information about both their paternal and maternal ancestries from 23 individuals. This allowed us to reconstruct a family tree in which a common ancestor, MP739, was identified (Fig. 3). The structure of the tree, alongside the fact that all parents of affected puppies were clinically normal, and that both males and females were affected, were all highly suggestive of an autosomal recessive mode of inheritance. During the study, complete information of all 42 cases was gathered. The complete family tree is shown in Supplementary file 1.

### Mapping of the critical region

**Fig. 4.**
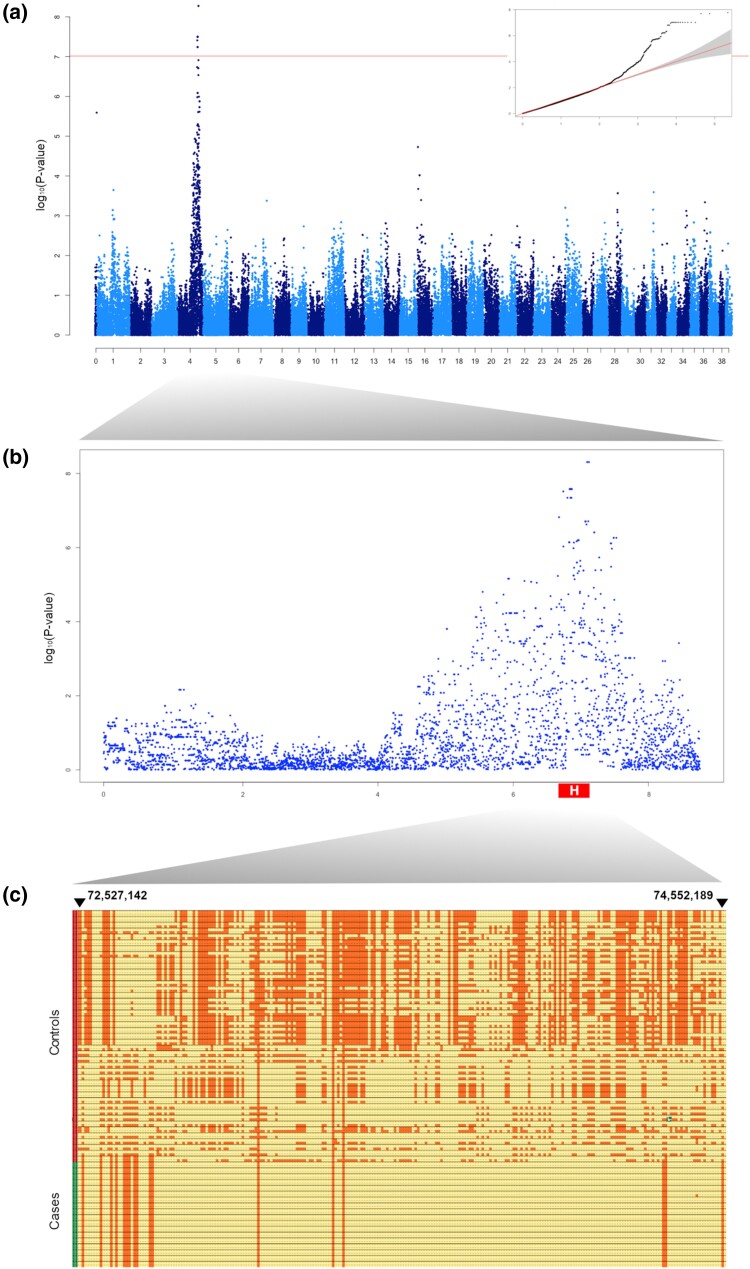
Genome-wide association analysis. a) Results of the GWAS obtained from analyzing the SNP chip data showing the negative log of the raw *P*-values calculated with the genotypic association test. The maximum value (minimum *P*-value) is =0.2.179e−08). Eighteen SNPs were above the Bonferroni correction threshold (horizontal line). In addition to the Manhattan plot, on top left the QQ-plot showing the observed (*y*-axis) vs expected (*x*-axis) quantile log *P*-values. Observe the distinct skewing confirming the strong association. b) Fine detail of the Manhattan plot showing CFA4. The critical region detected by homozygosity mapping is marked by the bold, white H. c) Detail of the associated region. Cases (lower left bar): 18 genotyped cases. Controls (upper left bar): the total of 41 controls. The boundaries of the homozygous region are marked.

To map a candidate genomic locus containing a putative causative variant, we carried out a GWAS. A cohort of 18 PWD cases and 20 PWD controls was genotyped on a 220k Illumina CanineHD BeadChip and used for the analysis. In addition, we merged the dataset with 23 normal PWD previously genotyped on a 170k Illumina CanineHD SNP chip, resulting in 171,159 SNPs shared between the two types of chips for the analysis. The merged dataset was then pruned for low genotyping rate (<95%), low minor allele frequency (<0.05), and Hardy–Weinberg equilibrium threshold (*P* < 10^−8^). After this filtering stage, 107,074 SNPs remained for the association analysis. The calculated genomic inflation factor (lambda) was equal to 1.09. We interpreted such lambda value as a sign of a very light stratification. We opted to use a mixed model for the association. Additionally, we used the strict Bonferroni correction (log 0.01/nr SNPs cutoff) to check the significance of the most associated SNPs. These reached an adjusted *P*-value of 2.178554e^−08^ and were located on CFA4. A total of 18 SNPs were above the Bonferroni correction. All these SNPs fell in an interval between CFA4: 71,186,169 (BICF2P428889) and CFA4: 74,034,481 (BICF2G630169846). All coordinates shown are Canfam4 (Fig. 4).

To define the interval containing the causal variant, we opted for a homozygosity mapping approach since the inheritance mechanism was indicated to be autosomal recessive, hypothesizing that affected dogs were most likely to be inbred. The homozygosity mapping pointed out a single homozygous region in CFA4 common in all the cases and not homozygous in the control group. We carefully examined the SNP data across the interval using a spreadsheet software, to define its boundaries. This region encompassed a ∼2 Mb interval in CFA4 between (Canfam4 converted coordinates) CFA4: 72,527,142 and CFA4: 74,552,389, using the last heterozygous markers before and after the homozygous segment.

### Whole-genome sequencing and variant detection

Taking all the information from the mapping strategies described the interval on CFA4 shared between all 18 cases was deemed the most likely to be associated with the cCMHD. Hence the search for disease variants was thereafter focused on the ∼2 Mb critical interval. We carried out whole-genome sequencing (WGS) using DNA from an affected dog.

**Fig. 5.**
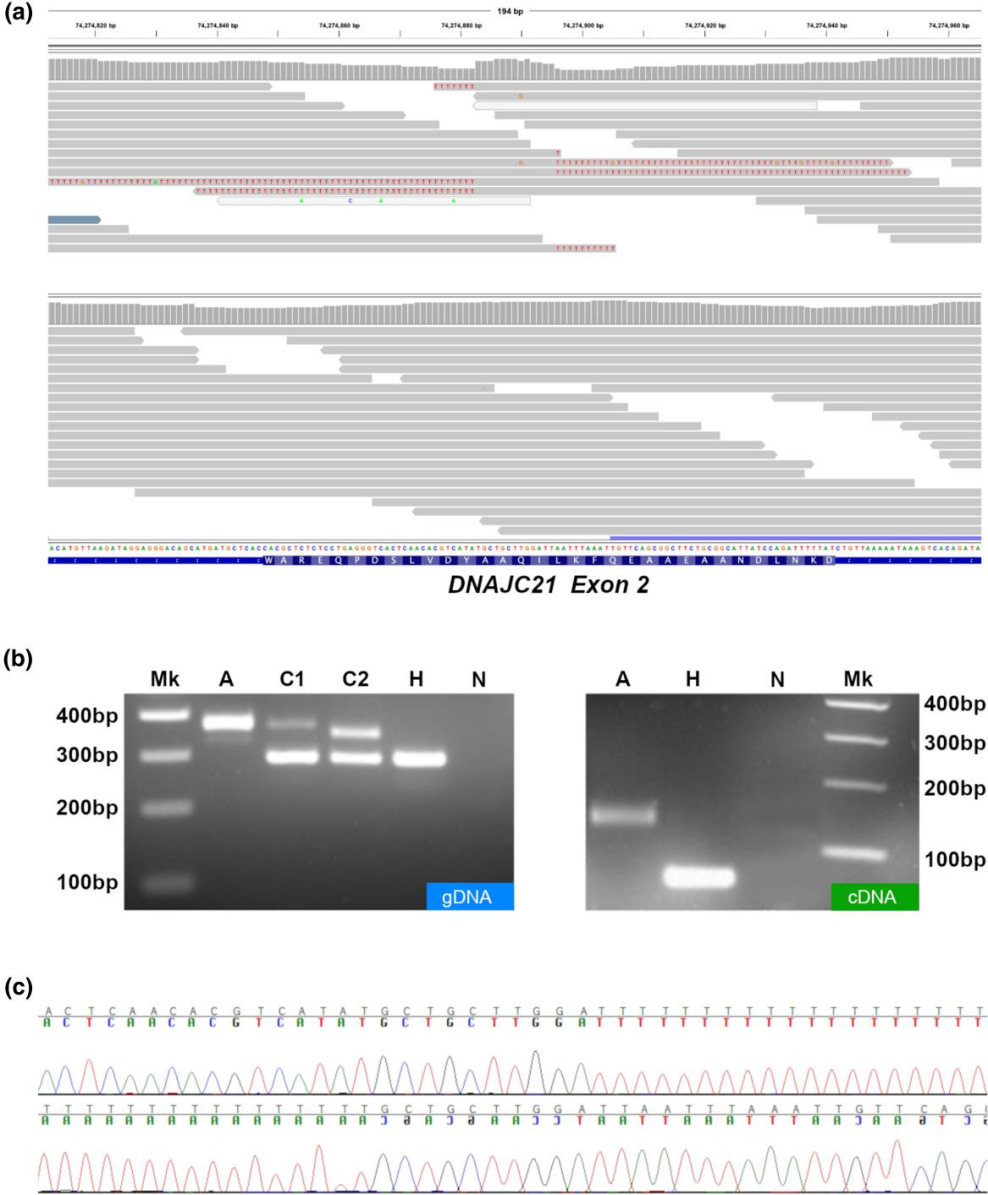
Sequencing and characterization of the mobile element. a) WGS of a case (top). Observe the poly-T sequences and the “bump” of coverage of the TGCTGCTTGGATT sequence. A control dog (internal database) is shown (bottom). b) Left: Electrophoresis gel of genomic DNA showing amplicons for a homozygous affected case (A) carriers (C1, C2) and a healthy wild type (H) and the negative control (N) are shown. Ladder (100 bp) noted as Mk. Note the differences in length of the mutant amplicon are due to variability in the polyT insertion. Right: Electrophoresis gel showing amplicons of the cDNA exon 2 amplification. Affected (A), healthy wild-type (H) and negative control (N). Ladder 100 bp in (Mk). Note the longer cDNA amplicon. c) Electropherograms of the Sanger sequencing of the mobile element insertion and the flaking region sequenced in both directions. Observe the repeated polyT.

Through filtering of the variants according to the assumed autosomal recessive inheritance, we found that 13,732 variants in the critical interval identified by WGS were homozygous in the affected dog. In parallel, variant calls resulting from WGS such as SNVs and small indels were filtered against the Dog10k database, and once converted to Canfam3.1, compared to the variants in the Dog Biomedical Variant Database Consortium (DBVDC), which are annotated using Canfam3.1. Notably, three of the dogs used for filtering were unaffected PWDs previously sequenced and examined by our group for a different project. A single SNV variant exclusive to the affected PWD was detected in the coding region of the *DNAJC1* gene, exon 2. The variant was initially reported as an insertion of a multiple T sequence by GATK, but upon closer inspection of the BAM file by IGV we identified the variant as a mobile element (see Fig. 5a). Subsequent PCR amplification (Fig. 5b) and genotyping assessed its segregation in the pedigree. The variant should therefore be described as (Canfam4) 74,274,883ins[T^70^]TGCTGCTTGGATT. Interestingly, genotyping of specific individuals revealed a variability in the length of the poly-T, with one to five additional bases occasionally sequenced. This could reflect truly existing length variation of the insertion allele in the PWD population or represent the consequence of technical artifacts during PCR and/or Sanger sequencing.

Real-time PCR-based mass genotyping of a larger PWD population found the variant in perfect linkage disequilibrium with the pathological condition described (Table 2). No large insertion, deletion, duplication or inversion associated with the disease was detected.

**Table 2.** Genotyped dogs.

Breed and availability of retinal phenotype	N OF DOGS	*[DEL*		
		wt/wt	wt/ins	Ins/Ins
Pwd—CASES	42	0	0	42
Pwd—Unaffected	1,000	950	50	0
Other breeds	1,204	1204	0	0
Total	2,246	2154	50	42

Dogs of other breeds belong to the consulted DBVDC and Dog10k databases. PWDs were genotyped as described in the *Methods* section.

Available blood-derived cDNA of a case was also extracted and compared to that of a control (Fig. 5b). Interestingly, the amplicon is larger (more than 100 bp) and contains a string of ∼50 A (reverse complement) followed by repeats of other bases in groups from ∼3 to ∼20.

### Nature of variant, position, and impact

The variant shows the typical repeat of the flanking target region of the mobile element (TGCTGCTTGGATT), plus a poly T of 70 bp of length (Fig. 5c)—essentially, a poly(T) stretch. The complete sequence, obtained through Sanger sequencing, is reported in Supplementary file 2.

Using available RNA-seq libraries generated in our laboratory from canine tissues, we re-annotated the dog *DNAJC21* gene, experimentally confirming the predicted transcript XM_038535356.1 (UU_Cfam_GSD_1.0 Canfam4—exons are reported in Supplementary File 2-1, and transcript Supplementary File 2-2). The translated protein (Supplementary File 2-3) aligns with the human DNAJC21 isoform (NP_001012339.2). The Clustal Omega alignment is reported in Supplementary File 2-4. The predicted protein was also BLASTed against a human reference with a 97% identity score.

**Fig. 6.**
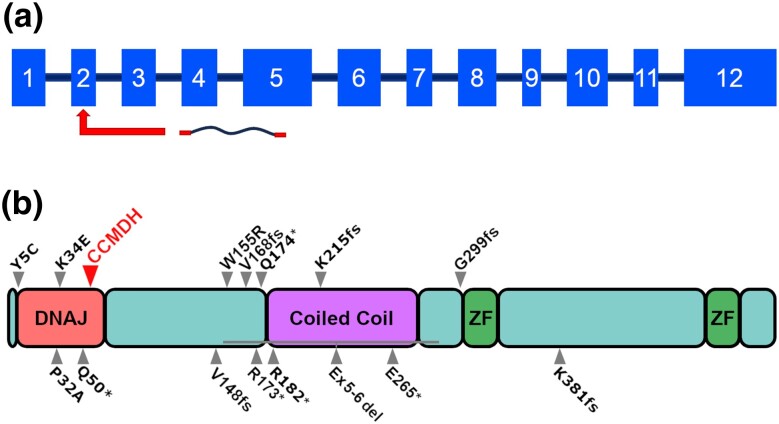
Impact of the genetic variant. a) Schematic of the DNAJC21 gene. The point of insertion in Exon 2 is indicated, observably very early in the predicted transcript. b) Larger arrowhead, position of the CCMDH-associated variant in the human DNAJC21 protein. Notably, the variant occurs at position 52, falling within the DNAJ domain. Smaller arrows: position of other DNAJC21 protein mutations in humans associated with BMF syndromes and (p.W155R, outside of the DNAJ domain) with syndromic microphthalmia. Variants are reported as described in literature (Tummala *et al.* 2016; D'Amours *et al*. 2018; Alsavaf *et al*. 2022; Feurstein 2023).

The variant is predicted to be inserted in exon 2 (Fig. 6a, Supplementary File 2-5). The predicted transcript with the most common polyA repeat insertion (reverse strand gene) of 70 bases (c.155insA^70^AATCCAAGCAGC; Supplementary File 2-6). If inserted in a sequence translator (https://web.expasy.org/translate/, accessed 06/22/2023), the predicted outcome is the synthesis of an aberrant product with a poly Lysine chain (K^23^), for additional amino acids and a premature stop codon (p.52fs28stop, Supplementary File 2-7). The variant occurs within the DNAJ domain of the DNAJC21 protein (Fig. 6b).

## Discussion

Through meticulous phenotyping and clinical analysis, combining multiple SNP chip-based mapping methods and WGS, we described and molecularly characterized an exonic poly-A insertion in *DNAJ21.* It is likely that the variant can be attributed to a mobile element insertional event, but it is extremely difficult to assess whether the remaining part of the element was deleted during the integration event or if a full-length SINE had integrated and subsequently lost most of its sequence in a second mutational event. If the latter event happened, it was not detected by our population genotyping.

The variant is consistently homozygous in all the cases of cCMHD and was not present in any the controls. A newly recognized disorder in PWDs, cCMHD is defined by complex eye malformations in association with hematologic and nonhematologic abnormalities. While the disease is inherited in an autosomal recessive manner, there are more affected females than there are males. This raises the question whether males are more likely to die before birth or be stillborn, and therefore being undetected.

Mobile element insertions can be highly deleterious, and there are several mechanisms by which new insertions have led to monogenic diseases (Payer and Burns 2019). In humans, the non-LTR retrotransposon Long Interspersed Element-1 (LINE-1 or L1) is the only active autonomous transposing element. To date, 124 LINE-1-mediated insertions that result in genetic diseases have been reported in humans (Hancks and Kazazian 2016). Short interspersed elements (SINEs) are also a major source of canine genomic diversity (Wang and Kirkness 2005). In fact, a large fraction of the dog genome has resulted from the expansion of transposable elements belonging to the SINE and long interspersed element (LINE) families. Molecular mapping has implicated mobile element insertions and retrogene insertions as the putative causal genetic variants underlying morphological differences (including selectively bred phenotypes) and inherited diseases (Lin *et al.* 1999; Brooks *et al.* 2003; Pele *et al.* 2005; Clark *et al.* 2006; Sutter *et al.* 2007; Credille *et al.* 2009; Parker *et al.* 2009; Downs and Mellersh 2014; Wolf *et al.* 2014; Brown *et al.* 2017; Marchant *et al.* 2017). Hitti-Marlin and colleagues mapped a LINE-1 insertion upstream of the *IMPG2* gene in a canine retinal disease (Hitti-Malin *et al.* 2020).

In our case, the type of element is most likely a SINE element reversely oriented and for which TGCTGCTTGGATT contains the target sequence. The presence of only the poly(T) is in our opinion not dissimilar to what has been described for a mutation event, UAB-R75103, in the work of Wimmer and colleagues, in which the insert is a poly(T) flanked by six bases (albeit in the variant described here, there is no deletion of the original sequence and loss of exonic sequence, but exclusively an insertion—(Wimmer *et al.* 2011) and an insertion variant containing a poly(A)29 tract as studied by one of the authors (Miyadera *et al.* 2012)). The *RAB3GAP1* SINE insertion described in Alaskan Huskies with a Warburg-like syndrome, which includes microphthalmia (Wiedmer *et al.* 2015) results in part of the affected exon being spliced out as an intron, but this is not the case of our insertion.

Albeit the additional bases in the cDNA amplicon could be an effect of the RNA synthesis, they are most probably an artifact of the enzyme-based repeated amplification process (i.e. RT-PCR).

The predicted outcome of the premature stop codon after the poly-lysine amino acid chain (p.52fs28stop) for the most common A^70^ transcript suggests a devastating effect on the N-terminus of the protein. Additionally, we can speculate that even in case of an altered reading frame due to the variability in the polyA repeats (A^71–75^ transcripts), the polyA would have a termination effect on the overall translation.

*DNAJC21* is a gene encoding a protein member of the DNAJ heat shock protein 40 family. DnaJ/Hsp40 (heat shock protein 40) proteins have been highly conserved through evolution and are important for protein translation, folding, unfolding, translocation, and degradation. They have a role in stimulating the ATPase activity of Hsp70 chaperones (Qiu *et al.* 2006). DNAJ21 has two N-terminal tetratricopeptide repeat domains and a C-terminal DNAJ domain. This protein binds the 45S pre-ribosomal RNA and may have a role in the biogenesis of the early nuclear ribosomal RNA, and also in the maturation of cytoplasmic pre-60S. DNAJC21 is a cofactor for the release of PA2G4 (cell cycle protein) and EBP1 (cell survival and differentiation) by ZNF622 (regulator of apoptosis) and HSPA1A (protein folding, ubiquitinase pathway) (Dorner *et al.* 2023).

Association of heat-shock protein genetic variants and microphthalmia has been reported in murine models carrying *HSPB4* variants with lens opacity, more severe in the homozygous form, which manifested as severe microphthalmia, detected at birth (Xi *et al.* 2008; Andley and Goldman 2016). In humans, frameshift *HSPB4* de novo mutations are reported in patients suffering from congenital aphakia, which is a developmental defect characterized by lens absence, microphthalmia, microcornea, and iris hypoplasia/aniridia (Marakhonov *et al.* 2020; Tedesco *et al.* 2022).

Biallelic variants in *DNAJC21* are mostly found to be associated in humans with bone marrow failure (BMF) syndrome type 3, with a phenotype that has a partial or complete overlap with Fanconi anemia, dyskeratosis congenita, Shwachman–Diamond syndrome (Dhanraj *et al.* 2017) and Diamond–Blackfan anemia (Chirita-Emandi *et al.* 2022). Interestingly, ocular manifestations in Fanconi and dyskeratosis congenita, which are ribosomopathies—inherited diseases in which the affected genes encode factors known to have a role in the synthesis of ribosomes—have been reported (Tsilou *et al.* 2010; De Keersmaecker *et al.* 2015; Aspesi and Ellis 2019).

Patients with global BMF can be prone to hematological cancers (Tummala *et al.* 2016). Some manifestations are overall consistent in most BMF patients: BMF, growth retardation, failure to thrive, developmental delay, recurrent infections, and skin, tooth or hair abnormalities. Nonetheless, specific individuals may manifest additional symptoms such as pancreatic insufficiency, liver cirrhosis, skeletal abnormalities, cryptorchidism, and others, and it has been suggested that increased reporting of clinical signs may be required to fully characterize the complete phenotypic spectrum (D'Amours *et al.* 2018). Ocular phenotypes are reported in relation with *DNAJC21*, with varied phenotypes including fundic lesions (rod–cone retinal dystrophy, foveal hypoplasia, choroidal thinning at the nasal extremity, altered myelination in the optic nerves), high myopia, and astigmatism (Tummala *et al.* 2016; Dhanraj *et al.* 2017; D'Amours *et al.* 2018; Chirita-Emandi *et al.* 2022; Dorner *et al.* 2023). Overall, previous works indicate that *DNAJC21* has important role in ocular morphology. The syndromic nature of defects in *DNAJC21* point out the need to fully clinically characterize patients with BMF, including a thorough ophthalmic examination (Tedesco *et al.* 2022).

Strikingly, in a case report, Alsavaf and colleagues report a 13.5-year-old female with thrombocytopenia and microdontia; she had a triangular face with microphthalmia (Alsavaf *et al.* 2022). The authors note that the DNAJC21 variant (W155R) they report falls outside the DNAJ domain. In our case, observing the alignment of the predicted canine protein with a human isoform and the available literature, the variant we describe seems to fall within the DNAJ domain. Dhanraj and colleagues describe a large diversity of loci for the *DNAJC21* variants associated with Shwachman–Diamond: Missense mutations are found in the extreme N-terminus (P32A and K34E) and premature stop codons or frameshifts occurring before the C-terminal zinc finger domains (R173X, Q174X, E265X; V148Kfs*30, G299Afs*2) (Dhanraj *et al.* 2017; Godley 2017; Feurstein 2023).

Overall, the position and nature of the variant seem to play only part of the role in the development of specific clinical manifestation. We hope that the canine model we propose can contribute to the search for ophthalmological manifestations and the development of specific treatments in human patients.

## Conclusions

GWAS and homozygosity mapping combined with WGS allowed us to detect an exonic mobile element insertion in *DNAJ21.* The variant is perfectly associated with the syndromic disorder, cCMHD, in the PWD, which is inherited as an autosomal recessive trait. cCMHD is characterized by complex eye malformations in association with hematologic and non-hematologic abnormalities. Our discovery adds to the list of canine ocular and systemic manifestations associated with a mobile element genetic variant. This spontaneous canine model constitutes a valuable starting point toward expansion of *DNAJ21*-associated genetic variants as well as mechanistic studies of the developmental abnormalities, and the potential development of specific therapies for this group of human multisystemic conditions.

## Supplementary Material

jkae067_Supplementary_Data

## Data Availability

Supplementary File 1 shows the whole 42 affected PWDs family tree. Supplementary File 2 contains the reannotated exons (2-1) and predicted transcript (2-2), and predicted protein (2-3), aligned with the human isoform NP_001012339.2 (2-4). The predicted polyT insertion sequence in (2-5); the predicted mutant DNAJC21 transcript in (2-6) and the predicted, truncated DNAJC21 protein in (2-7). The PWD SNP chip data generated for this paper (18 cases, 20 controls) and WGS data of case MP021 have been deposited to Dryad (https://datadryad.org/, accessed 06/22/2023), doi: 10.5061/dryad.z08kprrk3, link: https://datadryad.org/stash/share/jHZNCh1uPBy7pKYSg12IXN6w5HXRgi3Mq99vGC5FXLc. Supplemental material available at G3 online.

## References

[jkae067-B1] Acland GM, Aguirre GD, Ray J, Zhang Q, Aleman TS, Cideciyan AV, Pearce-Kelling SE, Anand V, Zeng Y, Maguire AM, et al 2001. Gene therapy restores vision in a canine model of childhood blindness. Nat Genet. 28(1):92–95. doi:10.1038/ng0501-92. 11326284

[jkae067-B2] Alsavaf MB, Verboon JM, Dogan ME, Azizoglu ZB, Okus FZ, Ozcan A, Dundar M, Eken A, Donmez-Altuntas H, Sankaran VG, et al 2022. A novel missense mutation outside the DNAJ domain of DNAJC21 is associated with Shwachman-Diamond syndrome. Br J Haematol. 197(6):e88–e93. doi:10.1111/bjh.18112. 35298850

[jkae067-B3] Andley UP, Goldman JW. 2016. Autophagy and UPR in alpha-crystallin mutant knock-in mouse models of hereditary cataracts. Biochim Biophys Acta. 1860(1):234–239. doi:10.1016/j.bbagen.2015.06.001. 26071686 PMC4673011

[jkae067-B4] Aspesi A, Ellis SR. 2019. Rare ribosomopathies: insights into mechanisms of cancer. Nat Rev Cancer. 19(4):228–238. doi:10.1038/s41568-019-0105-0. 30670820

[jkae067-B5] Aulchenko YS, Ripke S, Isaacs A, van Duijn CM. 2007. GenABEL: an R library for genome-wide association analysis. Bioinformatics. 23(10):1294–1296. doi:10.1093/bioinformatics/btm108. 17384015

[jkae067-B6] Beltran WA, Cideciyan AV, Lewin AS, Iwabe S, Khanna H, Sumaroka A, Chiodo VA, Fajardo DS, Román AJ, Deng WT, et al 2012. Gene therapy rescues photoreceptor blindness in dogs and paves the way for treating human X-linked retinitis pigmentosa. Proc Natl Acad Sci U S A. 109(6):2132–2137. doi:10.1073/pnas.1118847109. 22308428 PMC3277562

[jkae067-B7] Brooks MB, Gu W, Barnas JL, Ray J, Ray K. 2003. A line 1 insertion in the factor IX gene segregates with mild hemophilia B in dogs. Mamm Genome. 14(11):788–795. doi:10.1007/s00335-003-2290-z. 14722728

[jkae067-B8] Brown EA, Dickinson PJ, Mansour T, Sturges BK, Aguilar M, Young AE, Korff C, Lind J, Ettinger CL, Varon S, et al 2017. FGF4 retrogene on CFA12 is responsible for chondrodystrophy and intervertebral disc disease in dogs. Proc Natl Acad Sci U S A. 114(43):11476–11481. doi:10.1073/pnas.1709082114. 29073074 PMC5664524

[jkae067-B9] Chang CC, Chow CC, Tellier LC, Vattikuti S, Purcell SM, Lee JJ. 2015. Second-generation PLINK: rising to the challenge of larger and richer datasets. GigaScience. 4(1): 7. doi:10.1186/s13742-015-0047-8. 25722852 PMC4342193

[jkae067-B10] Chase K, Carrier DR, Adler FR, Ostrander EA, Lark KG. 2005. Interaction between the X chromosome and an autosome regulates size sexual dimorphism in Portuguese water dogs. Genome Res. 15(12):1820–1824. doi:10.1101/gr.3712705. 16339380 PMC1356120

[jkae067-B11] Chase K, Sargan D, Miller K, Ostrander EA, Lark KG. 2006. Understanding the genetics of autoimmune disease: two loci that regulate late onset Addison's disease in Portuguese water dogs. Int J Immunogenet. 33(3):179–184. doi:10.1111/j.1744-313X.2006.00593.x. 16712648 PMC2775482

[jkae067-B12] Chirita-Emandi A, Petrescu CA, Zimbru CG, Stoica F, Marian C, Ciubotaru A, Bataneant M, Puiu M. 2022. Case report: novel biallelic variants in DNAJC21 causing an inherited bone marrow failure spectrum phenotype: an odyssey to diagnosis. Front Genet. 13:870233. doi:10.3389/fgene.2022.870233. 35464845 PMC9023866

[jkae067-B13] Cingolani P, Platts A, Wang le L, Coon M, Nguyen T, Wang L, Land SJ, Lu X, Ruden DM. 2012. A program for annotating and predicting the effects of single nucleotide polymorphisms, SnpEff: SNPs in the genome of Drosophila melanogaster strain w1118; iso-2; iso-3. Fly (Austin). 6 (2):80–92. doi:10.4161/fly.19695. 22728672 PMC3679285

[jkae067-B14] Clark LA, Wahl JM, Rees CA, Murphy KE. 2006. Retrotransposon insertion in SILV is responsible for merle patterning of the domestic dog. Proc Natl Acad Sci U S A. 103(5):1376–1381. doi:10.1073/pnas.0506940103. 16407134 PMC1360527

[jkae067-B15] Credille KM, Minor JS, Barnhart KF, Lee E, Cox ML, Tucker KA, Diegel KL, Venta PJ, Hohl D, Huber M, et al 2009. Transglutaminase 1-deficient recessive lamellar ichthyosis associated with a LINE-1 insertion in Jack Russell terrier dogs. Br J Dermatol. 161(2):265–272. doi:10.1111/j.1365-2133.2009.09161.x. 19438474

[jkae067-B16] D'Amours G, Lopes F, Gauthier J, Saillour V, Nassif C, Wynn R, Alos N, Leblanc T, Capri Y, Nizard S, et al 2018. Refining the phenotype associated with biallelic DNAJC21 mutations. Clin Genet. 94(2):252–258. doi:10.1111/cge.13370. 29700810

[jkae067-B17] De Keersmaecker K, Sulima SO, Dinman JD. 2015. Ribosomopathies and the paradox of cellular hypo- to hyperproliferation. Blood. 125(9):1377–1382. doi:10.1182/blood-2014-10-569616. 25575543 PMC4342353

[jkae067-B18] Dell M . 2010. Severe bilateral microphthalmos in a Pomeranian pup. Can Vet J. 51(12):1405–1407. 21358938 PMC2978999

[jkae067-B19] Dhanraj S, Matveev A, Li H, Lauhasurayotin S, Jardine L, Cada M, Zlateska B, Tailor CS, Zhou J, Mendoza-Londono R, et al 2017. Biallelic mutations in DNAJC21 cause Shwachman-Diamond syndrome. Blood. 129(11):1557–1562. doi:10.1182/blood-2016-08-735431. 28062395

[jkae067-B20] Dorner K, Ruggeri C, Zemp I, Kutay U. 2023. Ribosome biogenesis factors—from names to functions. EMBO J. 42(7):e112699. doi:10.15252/embj.2022112699. 36762427 PMC10068337

[jkae067-B21] Downs LM, Mellersh CS. 2014. An intronic SINE insertion in FAM161A that causes exon-skipping is associated with progressive retinal atrophy in Tibetan Spaniels and Tibetan Terriers. PLoS One. 9(4):e93990. doi:10.1371/journal.pone.0093990. 24705771 PMC3976383

[jkae067-B22] Feurstein S . 2023. Emerging bone marrow failure syndromes—new pieces to an unsolved puzzle. Front Oncol. 13:1128533. doi:10.3389/fonc.2023.1128533. 37091189 PMC10119586

[jkae067-B23] Geiger DA, Miller AD, Cutter-Schatzberg K, Shelton GD, de Lahunta A, Schatzberg SJ. 2009. Encephalomyelopathy and polyneuropathy associated with neuronal vacuolation in two Boxer littermates. Vet Pathol. 46(6):1160–1165. doi:10.1354/vp.09-VP-0010-S-FL. 19605909

[jkae067-B24] Gelatt KN . 2014. Essentials of Veterinary Ophthalmology. Ames. Iowa: John Wiley & Sons, Inc.

[jkae067-B25] Gelatt KN . 2020. Veterinary Ophthalmology, pp. 1 Online Resource. Hoboken, NJ: Wiley-Blackwell.

[jkae067-B26] Gelatt KN, Gum GG. 1981. Inheritance of primary glaucoma in the beagle. Am J Vet Res. 42(10):1691–1693. 7325430

[jkae067-B27] Godley LA . 2017. DNAJC21: the new kid on the SDS block. Blood. 129(11):1413–1414. doi:10.1182/blood-2017-01-761635. 28302692

[jkae067-B28] Goldstein O, Zangerl B, Pearce-Kelling S, Sidjanin DJ, Kijas JW, Felix J, Acland GM, Aguirre GD. 2006. Linkage disequilibrium mapping in domestic dog breeds narrows the progressive rod-cone degeneration interval and identifies ancestral disease-transmitting chromosome. Genomics. 88(5):541–550. doi:10.1016/j.ygeno.2006.05.013. 16859891 PMC4006154

[jkae067-B29] Gould D, McLellan GJ. 2014. Chapter 8: The orbit and globe in BSAVA Manual of Canine and Feline Ophthalmology. 3rd ed. p. 117–118.

[jkae067-B30] Guziewicz KE, Cideciyan AV, Beltran WA, Komáromy AM, Dufour VL, Swider M, Iwabe S, Sumaroka A, Kendrick BT, Ruthel G, et al 2018. BEST1 gene therapy corrects a diffuse retina-wide microdetachment modulated by light exposure. Proc Natl Acad Sci U S A. 115(12):E2839–E2848. doi:10.1073/pnas.1720662115. 29507198 PMC5866594

[jkae067-B31] Hancks DC, Kazazian HH Jr. 2016. Roles for retrotransposon insertions in human disease. Mob DNA. 7(1):9. doi:10.1186/s13100-016-0065-9. 27158268 PMC4859970

[jkae067-B32] Hitti-Malin RJ, Burmeister LM, Ricketts SL, Lewis TW, Pettitt L, Boursnell M, Schofield EC, Sargan D, Mellersh CS. 2020. A LINE-1 insertion situated in the promoter of IMPG2 is associated with autosomal recessive progressive retinal atrophy in Lhasa Apso dogs. BMC Genet. 21(1):100. doi:10.1186/s12863-020-00911-w. 32894063 PMC7487703

[jkae067-B33] Hyttel P . 2010. Essentials of Domestic Animal Embryology. Edinburgh; New York: Saunders/Elsevier.

[jkae067-B34] Jagannathan V, Drogemuller C, Leeb T; Dog Biomedical Variant Database Consortium. 2019. A comprehensive biomedical variant catalogue based on whole genome sequences of 582 dogs and eight wolves. Anim Genet. 50(6):695–704. doi:10.1111/age.12834. 31486122 PMC6842318

[jkae067-B35] Kaukonen M, Woods S, Ahonen S, Lemberg S, Hellman M, Hytönen MK, Permi P, Glaser T, Lohi H. 2018. Maternal inheritance of a recessive RBP4 defect in canine congenital eye disease. Cell Rep. 23(9):2643–2652. doi:10.1016/j.celrep.2018.04.118. 29847795 PMC6546432

[jkae067-B36] Komáromy AM, Alexander JJ, Rowlan JS, Garcia MM, Chiodo VA, Kaya A, Tanaka JC, Acland GM, Hauswirth WW, Aguirre GD. 2010. Gene therapy rescues cone function in congenital achromatopsia. Hum Mol Genet. 19(13):2581–2593. doi:10.1093/hmg/ddq136. 20378608 PMC2883338

[jkae067-B37] Laratta LJ, Riis RC, Kern TJ, Koch SA. 1985. Multiple congenital ocular defects in the Akita dog. Cornell Vet. 75(3):381–392. 3926378

[jkae067-B38] Li H . 2011. A statistical framework for SNP calling, mutation discovery, association mapping and population genetical parameter estimation from sequencing data. Bioinformatics. 27(21):2987–2993. doi:10.1093/bioinformatics/btr509. 21903627 PMC3198575

[jkae067-B39] Li H, Durbin R. 2009. Fast and accurate short read alignment with Burrows-Wheeler transform. Bioinformatics. 25(14):1754–1760. doi:10.1093/bioinformatics/btp324. 19451168 PMC2705234

[jkae067-B40] Li H, Handsaker B, Wysoker A, Fennell T, Ruan J, Homer N, Marth G, Abecasis G, Durbin R; 1000 Genome Project Data Processing Subgroup. 2009. The sequence alignment/map format and SAMtools. Bioinformatics. 25(16):2078–2079. doi:10.1093/bioinformatics/btp352. 19505943 PMC2723002

[jkae067-B41] Lin L, Faraco J, Li R, Kadotani H, Rogers W, Lin X, Qiu X, de Jong PJ, Nishino S, Mignot E. 1999. The sleep disorder canine narcolepsy is caused by a mutation in the hypocretin (orexin) receptor 2 gene. Cell. 98(3):365–376. doi:10.1016/S0092-8674(00)81965-0. 10458611

[jkae067-B42] Llorente-Gonzalez S, Peralta-Calvo J, Abelairas-Gomez JM. 2011. Congenital anophthalmia and microphthalmia: epidemiology and orbitofacial rehabilitation. Clin Ophthalmol. 5:1759–1765. doi:10.2147/OPTH.S27189. 22267908 PMC3258083

[jkae067-B43] Marakhonov AV, Voskresenskaya AA, Ballesta MJ, Konovalov FA, Vasilyeva TA, Blanco-Kelly F, Pozdeyeva NA, Kadyshev VV, López-González V, Guillen E, et al 2020. Expanding the phenotype of CRYAA nucleotide variants to a complex presentation of anterior segment dysgenesis. Orphanet J Rare Dis. 15(1):207. doi:10.1186/s13023-020-01484-8. 32791987 PMC7427288

[jkae067-B44] Marchant TW, Johnson EJ, McTeir L, Johnson CI, Gow A, Liuti T, Kuehn D, Svenson K, Bermingham ML, Drögemüller M, et al 2017. Canine brachycephaly is associated with a retrotransposon-mediated missplicing of SMOC2. Curr Biol. 27(11):1573–1584.e1576. doi:10.1016/j.cub.2017.04.057. 28552356 PMC5462623

[jkae067-B45] McKenna A, Hanna M, Banks E, Sivachenko A, Cibulskis K, Kernytsky A, Garimella K, Altshuler D, Gabriel S, Daly M, et al 2010. The genome analysis toolkit: a MapReduce framework for analyzing next-generation DNA sequencing data. Genome Res. 20(9):1297–1303. doi:10.1101/gr.107524.110. 20644199 PMC2928508

[jkae067-B46] Mellersh CS . 2014. The genetics of eye disorders in the dog. Canine Genet Epidemiol. 1(1):3. doi:10.1186/2052-6687-1-3. 26401320 PMC4574392

[jkae067-B47] Mhlanga-Mutangadura T, Johnson GS, Ashwini A, Shelton GD, Wennogle SA, Johnson GC, Kuroki K, O'Brien DP. 2016. A homozygous RAB3GAP1:c.743delC mutation in rottweilers with neuronal vacuolation and spinocerebellar degeneration. J Vet Intern Med. 30(3):813–818. doi:10.1111/jvim.13921. 26968732 PMC4913561

[jkae067-B48] Mhlanga-Mutangadura T, Johnson GS, Schnabel RD, Taylor JF, Johnson GC, Katz ML, Shelton GD, Lever TE, Giuliano E, Granger N, et al 2016. A mutation in the Warburg syndrome gene, RAB3GAP1, causes a similar syndrome with polyneuropathy and neuronal vacuolation in black Russian terrier dogs. Neurobiol Dis. 86:75–85. doi:10.1016/j.nbd.2015.11.016. 26607784

[jkae067-B49] Miyadera K, Brierley I, Aguirre-Hernandez J, Mellersh CS, Sargan DR. 2012. Multiple mechanisms contribute to leakiness of a frameshift mutation in canine cone-rod dystrophy. PLoS One. 7(12):e51598. doi:10.1371/journal.pone.0051598. 23251588 PMC3520932

[jkae067-B50] Miyadera K, Santana E, Roszak K, Iffrig S, Visel M, Iwabe S, Boyd RF, Bartoe JT, Sato Y, Gray A, et al 2022. Targeting ON-bipolar cells by AAV gene therapy stably reverses LRIT3-congenital stationary night blindness. Proc Natl Acad Sci U S A. 119(13):e2117038119. doi:10.1073/pnas.2117038119. PMC9060458 35316139

[jkae067-B51] Murgiano L, Becker D, Spector C, Carlin K, Santana E, Niggel JK, Jagannathan V, Leeb T, Pearce-Kelling S, Aguirre GD, et al 2020. CCDC66 frameshift variant associated with a new form of early-onset progressive retinal atrophy in Portuguese water dogs. Sci Rep. 10(1):21162. doi:10.1038/s41598-020-77980-5. 33273526 PMC7712861

[jkae067-B52] Parker HG, VonHoldt BM, Quignon P, Margulies EH, Shao S, Mosher DS, Spady TC, Elkahloun A, Cargill M, Jones PG, et al 2009. An expressed fgf4 retrogene is associated with breed-defining chondrodysplasia in domestic dogs. Science. 325(5943):995–998. doi:10.1126/science.1173275. 19608863 PMC2748762

[jkae067-B53] Payer LM, Burns KH. 2019. Transposable elements in human genetic disease. Nat Rev Genet. 20(12):760–772. doi:10.1038/s41576-019-0165-8. 31515540

[jkae067-B54] Peiffer RL Jr, Fischer CA. 1983. Microphthalmia, retinal dysplasia, and anterior segment dysgenesis in a litter of Doberman Pinschers. J Am Vet Med Assoc. 183(8):875–878. 6415022

[jkae067-B55] Pele M, Tiret L, Kessler JL, Blot S, Panthier JJ. 2005. SINE exonic insertion in the PTPLA gene leads to multiple splicing defects and segregates with the autosomal recessive centronuclear myopathy in dogs. Hum Mol Genet. 14(11):1417–1427. doi:10.1093/hmg/ddi151. 15829503

[jkae067-B56] Qiu XB, Shao YM, Miao S, Wang L. 2006. The diversity of the DnaJ/Hsp40 family, the crucial partners for Hsp70 chaperones. Cell Mol Life Sci. 63(22):2560–2570. doi:10.1007/s00018-006-6192-6. 16952052 PMC11136209

[jkae067-B57] Rausch T, Zichner T, Schlattl A, Stütz AM, Benes V, Korbel JO. 2012. DELLY: structural variant discovery by integrated paired-end and split-read analysis. Bioinformatics. 28(18):i333–i339. doi:10.1093/bioinformatics/bts378. 22962449 PMC3436805

[jkae067-B58] Reis LM, Semina EV. 2011. Genetics of anterior segment dysgenesis disorders. Curr Opin Ophthalmol. 22(5):314–324. doi:10.1097/ICU.0b013e328349412b. 21730847 PMC3558283

[jkae067-B59] Saraiva IQ, Delgado E. 2020. Congenital ocular malformations in dogs and cats: 123 cases. Vet Ophthalmol. 23(6):964–978. doi:10.1111/vop.12836. 33058381

[jkae067-B60] Saunders GK, Wood PA, Myers RK, Shell LG, Carithers R. 1988. GM1 gangliosidosis in Portuguese water dogs: pathologic and biochemical findings. Vet Pathol. 25(4):265–269. doi:10.1177/030098588802500403. 3136586

[jkae067-B61] Searle A, Shetty P, Melov SJ, Alahakoon TI. 2018. Prenatal diagnosis and implications of microphthalmia and anophthalmia with a review of current ultrasound guidelines: two case reports. J Med Case Rep. 12(1):250. doi:10.1186/s13256-018-1746-4. 30153864 PMC6114735

[jkae067-B62] Shaw GC, Tse MPY, Miller AD. 2019. Microphthalmia with multiple anterior segment defects in Portuguese water dogs. Vet Pathol. 56(2):269–273. doi:10.1177/0300985818794248. 30131012

[jkae067-B63] Sinnwell JP, Therneau TM, Schaid DJ. 2014. The kinship2 R package for pedigree data. Hum Hered. 78(2):91–93. doi:10.1159/000363105. 25074474 PMC4154601

[jkae067-B64] Slatter DH . 1990. Fundamentals of Veterinary Ophthalmology. Philadelphia, PA: Saunders.

[jkae067-B65] Sutter NB, Bustamante CD, Chase K, Gray MM, Zhao K, Zhu L, Padhukasahasram B, Karlins E, Davis S, Jones PG, et al 2007. A single IGF1 allele is a major determinant of small size in dogs. Science. 316(5821):112–115. doi:10.1126/science.1137045. 17412960 PMC2789551

[jkae067-B66] Tedesco B, Cristofani R, Ferrari V, Cozzi M, Rusmini P, Casarotto E, Chierichetti M, Mina F, Galbiati M, Piccolella M, et al 2022. Insights on human small heat shock proteins and their alterations in diseases. Front Mol Biosci. 9:842149. doi:10.3389/fmolb.2022.842149. 35281256 PMC8913478

[jkae067-B67] Tsilou ET, Giri N, Weinstein S, Mueller C, Savage SA, Alter BP. 2010. Ocular and orbital manifestations of the inherited bone marrow failure syndromes: fanconi anemia and dyskeratosis congenita. Ophthalmology. 117(3):615–622. doi:10.1016/j.ophtha.2009.08.023. 20022637 PMC2830377

[jkae067-B68] Tummala H, Walne AJ, Williams M, Bockett N, Collopy L, Cardoso S, Ellison A, Wynn R, Leblanc T, Fitzgibbon J, et al 2016. DNAJC21 mutations link a cancer-prone bone marrow failure syndrome to corruption in 60S ribosome subunit maturation. Am J Hum Genet. 99(1):115–124. doi:10.1016/j.ajhg.2016.05.002. 27346687 PMC5005432

[jkae067-B69] Untergasser A, Cutcutache I, Koressaar T, Ye J, Faircloth BC, Remm M, Rozen SG. 2012. Primer3—new capabilities and interfaces. Nucleic Acids Res. 40(15):e115. doi:10.1093/nar/gks596. 22730293 PMC3424584

[jkae067-B70] Verma AS, Fitzpatrick DR. 2007. Anophthalmia and microphthalmia. Orphanet J Rare Dis. 2(1):47. doi:10.1186/1750-1172-2-47. 18039390 PMC2246098

[jkae067-B71] Wang R, Wiggs JL. 2014. Common and rare genetic risk factors for glaucoma. Cold Spring Harb Perspect Med. 4(12):a017244. doi:10.1101/cshperspect.a017244. 25237143 PMC4292091

[jkae067-B72] Wang W, Kirkness EF. 2005. Short interspersed elements (SINEs) are a major source of canine genomic diversity. Genome Res. 15(12):1798–1808. doi:10.1101/gr.3765505. 16339378 PMC1356118

[jkae067-B73] Wiedmer M, Oevermann A, Borer-Germann SE, Gorgas D, Shelton GD, Drögemüller M, Jagannathan V, Henke D, Leeb T. 2015. A RAB3GAP1 SINE insertion in Alaskan huskies with polyneuropathy, ocular abnormalities, and neuronal vacuolation (POANV) resembling human warburg micro syndrome 1 (WARBM1). G3 (Bethesda). 6 (2):255–262. doi:10.1534/g3.115.022707. 26596647 PMC4751546

[jkae067-B74] Wimmer K, Callens T, Wernstedt A, Messiaen L. 2011. The NF1 gene contains hotspots for L1 endonuclease-dependent de novo insertion. PLoS Genet. 7(11):e1002371. doi:10.1371/journal.pgen.1002371. 22125493 PMC3219598

[jkae067-B75] Wolf ZT, Leslie EJ, Arzi B, Jayashankar K, Karmi N, Jia Z, Rowland DJ, Young A, Safra N, Sliskovic S, et al 2014. A LINE-1 insertion in DLX6 is responsible for cleft palate and mandibular abnormalities in a canine model of Pierre Robin sequence. PLoS Genet. 10(4):e1004257. doi:10.1371/journal.pgen.1004257. 24699068 PMC3974639

[jkae067-B76] Xi JH, Bai F, Gross J, Townsend RR, Menko AS, Andley UP. 2008. Mechanism of small heat shock protein function in vivo: a knock-in mouse model demonstrates that the R49C mutation in alpha A-crystallin enhances protein insolubility and cell death. J Biol Chem. 283(9):5801–5814. doi:10.1074/jbc.M708704200. 18056999

[jkae067-B77] Zangerl B, Goldstein O, Philp AR, Lindauer SJ, Pearce-Kelling SE, Mullins RF, Graphodatsky AS, Ripoll D, Felix JS, Stone EM, et al 2006. Identical mutation in a novel retinal gene causes progressive rod–cone degeneration in dogs and retinitis pigmentosa in humans. Genomics. 88(5):551–563. doi:10.1016/j.ygeno.2006.07.007. 16938425 PMC3989879

[jkae067-B78] Zhang RL, Samuelson DA, Zhang ZG, Reddy VN, Shastry BS. 1991. Analysis of eye lens-specific genes in congenital hereditary cataracts and microphthalmia of the miniature schnauzer dog. Invest Ophthalmol Vis Sci. 32(9):2662–2665. 1869417

