# Supplementary material for: A naturally occurring canine model of syndromic congenital microphthalmia
Source: G3 (Bethesda). 2024 Apr 29;14(6):jkae067. doi: 10.1093/g3journal/jkae067 (PMC11152080; doi:10.1093/g3journal/jkae067)
Supplement: jkae067_Supplementary_Data [file jkae067_supplementary_data.zip › Supplementary_File_2_G3-2024-404875.pdf]

Supplementary File 2-1: Re-annotated *DNACJ1*

>Exon12
CTTCCACAATGCAATAAATTGCAGATCACCGAAATTTTAACTCTTTAGATGATTTCAGTTCAGTTTTTGGTTT
CAAAATCTAGAGACAATCAAAAAGAAAAAGTGTTGGCAGAATTTCTATCTGTTTTTACGTTTCTCTTTCTTG
CTTCGACTACTTGTTATGCTGCCTAAAGAACATGATGAAGGTGCTCTTGCATGACCTGTGGCTTTCAGATGG
TCGAAAAGTTTATTCCGGGATGGAAATTCACTATGGCAGGTTGTACAGCTGATAAGAACATCACT

>Exon11
CACTGTCTGTGGTTCAGCAGGTACTTTGACAGATTTTTTCGTATCTTTGGCTTTCTTTCCTTTGGGTTTAGG
AGCA

>Exon10
TTCTTAGCTTCACTTTTTGGATCATCACAGAGTTGGACAGTCTCTGTGACACTGCTATTTGCTTGGGGACTA
CCTTCTAGTTCTTTGGCACAGTCTTGATTTAAATTGGTATCTTCTGATTCAACCTTTACTCCTTCTTCAGTTCC
ATTTTCATTAAAATTATCATCATAATT

>Exon9
CTGTGCTGGTTTCTGTTTCTTTTTCTTCTGCTTTTTAGAAAGC

>Exon8
TTTTGTTTTGGTGCATCTTCTATTTCTTCCTCAGAATTGGCATTCAATGGATTTTCATCAGTTTGAGGTCCTG
AAAAATTTTCTTCCTCCTCCTCCAACTGTTGTTTTAACAAAGCAACCATTTCCCGATGCTTCTTTGATTTTTC
ATGATTCCTCATA

>Exon7
GCCTTTTCAGTCTTGAAAGATTTGTCACATGCTGGGCAGTAAAGGTCATCGTACAGCTCAGCATCCTCAGC
TTCATCACTGTCTTTAC

>Exon6
CTTCGTGCCCATCTTTGAGTTCAGATTCctccatctcctcttcatctgatccatctccaaactccttctcatagcgcgcctccatctc
cttgagctccttctccaGGTCGGCCACGGTCATCCAGCTCTGCTCTCTGTACTGCTCTGCCAGC

>Exon5
TTTGCCTGCTTCAGCTTCTGCTGCCGCCTCATCTCCTCTGCCTTCCTCGCCTTCTCAGCGTTCTGTTCCTCCA
CGAGCTTCCGATGCGCCTGCACTCTCCTGTCTCTTTTCCGGATGAACGCAACCAGCTGACGGACAAGCTCA
TTCTTCTCCTTCCTTGCTTTGTCGCGAATCTTTTTGTTTTCTTTTTCCATGGCTCGTTTCTCCCAGCGGTTTGA
AGCTTGTCGTGTATCATATTCTTCCTTCCAAGCAAAGCTCTTCTGAGTGCAGAAACTCTGCCAATAGGCATA
GAAAGGATGGACTAC

>Exon4
CGTATCATAGTCGCTCTGGGAGTCTCCAAAAGTGGGGAAATCTTCCATCTCCTCTTCTAACACAGATTCTAG
TTCTTCCTTCGCAATCATTTCAAAAACATTACGATAGACTGTATAAAAGCC

>Exon3
CTTTTCATCATCTCCATAACCAGAGTAACAGGTCACAGTGAAATAGTGAAGCAAATCTAAGCTGTCGTCTTG
ATATTCCCCATCAAGCCCACCTTTAAGTAGAGCTTCTCTATGATTATCATAC

>Exon2
CACGCTCTCTCCTGAGGGTCACTCAACACGTCATATGCTGCTTGGATTAATTTAAATTGTTCAGCGGCTTCT
GCGGCATTATCCAGATTTTTAT

>Exon1
CCGGGTGCCATTTCAGGGCCAGCTTCCGATAGGCCTTCTTGAGCTCCTCCTCGCTGGCGTCGCGCCGCACC
CCCAGCGCTTCGTAGTGACACTTCAT

Supplementary File 2-2: Coding transcript

>DNAJC21_Transcr_Dog
ATGAAGTGTCACTACGAAGCGCTGGGGGTGCGGCGCGACGCCAGCGAGGAGGAGCTCAAGAAGGCCTA
TCGGAAGCTGGCCCTGAAATGGCACCCGGATAAAAATCTGGATAATGCCGCAGAAGCCGCTGAACAATTT
AAATTAATCCAAGCAGCATATGACGTGTTGAGTGACCCTCAGGAGAGAGCGTGGTATGATAATCATAGAGA
AGCTCTACTTAAAGGTGGGCTTGATGGGGAATATCAAGACGACAGCTTAGATTTGCTTCACTATTTCACTGT
GACCTGTTACTCTGGTTATGGAGATGATGAAAAGGGCTTTTATACAGTCTATCGTAATGTTTTTGAAATGATT
GCGAAGGAAGAACTAGAATCTGTGTTAGAAGAGGAGATGGAAGATTTCCCCACTTTTGGAGACTCCCAG
AGCGACTATGATACGGTAGTCCATCCTTTCTATGCCTATTGGCAGAGTTTCTGCACTCAGAAGAGCTTTGCT
TGGAAGGAAGAATATGATACACGACAAGCTTCAAACCGCTGGGAGAAACGAGCCATGGAAAAAGAAAAC
AAAAAGATTCGCGACAAAGCAAGGAAGGAGAAGAATGAGCTTGTCCGTCAGCTGGTTGCGTTCATCCGG
AAAAGAGACAGGAGAGTGCAGGCGCATCGGAAGCTCGTGGAGGAACAGAACGCTGAGAAGGCGAGGA
AGGCAGAGGAGATGAGGCGGCAGCAGAAGCTGAAGCAGGCAAAGCTGGCAGAGCAGTACAGAGAGCA
GAGCTGGATGACCGTGGCCGACCtggagaaggagctcaaggagatggaggcgcgctatgagaaggagtttggagatggatca
gatgaagaggagatggagGAATCTGAACTCAAAGATGGGCACGAAGGTAAAGACAGTGATGAAGCTGAGGAT
GCTGAGCTGTACGATGACCTTTACTGCCCAGCATGTGACAAATCTTTCAAGACTGAAAAGGCTATGAGGAA
TCATGAAAAATCAAAGAAGCATCGGGAAATGGTTGCTTTGTTAAAACAACAGTTGGAGGAGGAGGAAGA
AAATTTTTCAGGACCTCAAACTGATGAAAATCCATTGAATGCCAATTCTGAGGAAGAAATAGAAGATGCAC
CAAAACAAAAGCTTTCTAAAAAGCAGAAGAAAAAGAAACAGAAACCAGCACAGAATTATGATGATAATTT
TAATGAAAATGGAACTGAAGAAGGAGTAAAGGTTGAATCAGAAGATACCAATTTAAATCAAGACTGTGCC
AAAGAACTAGAAGGTAGTCCCCAAGCAAATAGCAGTGTCACAGAGACTGTCCAACTCTGTGATGATCCAA
AAAGTGAAGCTAAGAATGCTCCTAAACCCAAAGGAAAGAAAGCCAAAGATACGAAAAAATCTGTCAAAG
TACCTGCTGAACCACAGACAGTGAGTGATGTTCTTATCAGCTGTACAACCTGCCATAGTGAATTTCCATCCC
GGAATAAACTTTTCGACCATCTGAAAGCCACAGGTCATGCAAGAGCACCTTCATCATGTTCTTTAGGCAGC
ATAACAAGTAGTCGAAGCAAGAAAGAGAAACGTAAAAACAGATAGAAATTCTGCCAACACTTTTTCTTTTT
GATTGTCTCTAGATTTTGAAACCAAAAACTGAACTGAAATCATCTAAAGAGTTAAAATTTCGGTGATCTGCA
ATTTATTGCATTGTGGAAG

Supplementary File 2-3: Predicted canine protein

>Predicted_Protein
MKCHYEALGVRRDASEEELKKAYRKLALKWHPDKNLDNAAEAAEQFKLIQAAYDVLSDPQERAWYDNHREA
LLKGGLDGEYQDDSLDLLHYFTVTCYSGYGDDEKGFYTVYRNVFEMIAKEELESVLEEEMEDFPTFGDSQSDY
DTVVHPFYAYWQSFCTQKSFAWKEEYDTRQASNRWEKRAMEKENKKIRDKARKEKNELVRQLVAFIRKRDR
RVQAHRKLVEEQNAEKARKAEEMRRQQKLKQAKLAEQYREQSWMTVADLEKELKEMEARYEKEFGDGSDE
EEMEESELKDGHEGKDSDEAEDAELYDDLYCPACDKSFKTEKAMRNHEKSKKHREMVALLKQQLEEEEENFS
GPQTDENPLNANSEEEIEDAPKQKLSKKQKKKKQKPAQNYDDNFNENGTEEGVKVESEDTNLNQDCAKELE
GSPQANSSVTETVQLCDDPKSEAKNAPKPGKKAKDTKKSVKVPAEPQTVSDVLISCTTCHSEFPSRNKLFDHL
KATGHARAPSSCSLGSITSSRSKKEKRKNR

Supplementary File 2-4: Alignment with human DNAJC21 protein

```
Predicted_Protein      MKCHYEALGVRRDASEEELKKAYRKLALKWHPDKNLDNAAEAAEQFKLIQAAYDVLSDPQ   60
NP_001012339.2         MKCHYEALGVRRDASEEELKKAYRKLALKWHPDKNLDNAAEAAEQFKLIQAAYDVLSDPQ   60
                       ************************************************************

Predicted_Protein      ERAWYDNHREALLKGGLDGEYQDDSLDLLHYFTVTCYSGYGDDEKGFYTVYRNVFEMIAK   120
NP_001012339.2         ERAWYDNHREALLKGGFDGEYQDDSLDLLRYFTVTCYSGYGDDEKGFYTVYRNVFEMIAK   120
                       ****************:************:******************************

Predicted_Protein      EELESVLEEEMEDFPTFGDSQSDYDTVVHPFYAYWQSFCTQKSFAWKEEYDTRQASNRWE   180
NP_001012339.2         EELESVLEEEVDDFPTFGDSQSDYDTVVHPFYAYWQSFCTQKNFAWKEEYDTRQASNRWE   180
                       **********::******************************.*****************

Predicted_Protein      KRAMEKENKKIRDKARKEKNELVRQLVAFIRKRDRRVQAHRKLVEEQNAEKARKAEEMRR   240
NP_001012339.2         KRAMEKENKKIRDKARKEKNELVRQLVAFIRKRDKRVQAHRKLVEEQNAEKARKAEEMRR   240
                       **********************************:*************************

Predicted_Protein      QQKLKQAKLAEQYREQSWMTVADLEKELKEMEARYEKEFGDGSDEEEMEESELKDGHEGK   300
NP_001012339.2         QQKLKQAKLVEQYREQSWMTMANLEKELQEMEARYEKEFGDGSDENEMEEHELKDEEDGK   300
                       *********.**********:*:*****:****************:**** **** .:**

Predicted_Protein      DSDEAEDAELYDDLYCPACDKSFKTEKAMRNHEKSKKHREMVALLKQQLEEEEENFSGPQ   360
NP_001012339.2         DSDEAEDAELYDDLYCPACDKSFKTEKAMKNHEKSKKHREMVALLKQQLEEEEENFSRPQ   360
                       *****************************:*************************** **

Predicted_Protein      TDENPLNANSEEEIEDAPKQKLSKKQKKKKQKPAQNYDDNFNENGTEEGVKVESEDTNLN   420
NP_001012339.2         IDENPLDDNSEEEMEDAPKQKLSKKQKKKKQKPAQNYDDNFNVNGPGEGVKVDPEDTNLN   420
                        *****: *****:**************************** **  *****: ******

Predicted_Protein      QDCAKELEGSPQANSSVTETVQLCDDPKSEAKNAPKPGKKAKDTKKSVKVPAEPQTVSD   480
NP_001012339.2         QDSAKELEDSPQENVSVTEIIKPCDDPKSEAKSVPKPGKKTKDMKKPVRVPAEPQTMS-   479
                       **.*****.*** * **** :: *********..*******:** ** *:*******:*

Predicted_Protein      VLISCTTCHSEFPSRNKLFDHLKATGHARAPSSCSLGSITSSRSKKEKRKNR 532
NP_001012339.2         VLISCTTCHSEFPSRNKLFDHLKATGHARAPSSSSLNSATSSQSKKEKRKNR 531
                       *********************************.**.* ***:*********
```

Supplementary File 2-5: predicted polyT insertion sequence

>PolyT_Insertion
TGCTGCTTGGATTTTTTTTTTTTTTTTTTTTTTTTTTTTTTTTTTTTTTTTTTTTTTTTTTTTTTTTTTTTTTTT
TTTTTTTTGCTGCTTGGATT

Supplementary File 2-6: Predicted mutant transcript

>Mutant transcript
ATGAAGTGTCACTACGAAGCGCTGGGGGTGCGGCGCGACGCCAGCGAGGAGGAGCTCAAGAAGGCCTA
TCGGAAGCTGGCCCTGAAATGGCACCCGGATAAAAATCTGGATAATGCCGCAGAAGCCGCTGAACAATTT
AAATTAATCCAAGCAGCAAAAAAAAAAAAAAAAAAAAAAAAAAAAAAAAAAAAAAAAAAAAAAAAAAA
AAAAAAAAAAAAAAAAAAAAATCCAAGCAGCATATGACGTGTTGAGTGACCCTCAGGAGAGAGCGTGGT
ATGATAATCATAGAGAAGCTCTACTTAAAGGTGGGCTTGATGGGGAATATCAAGACGACAGCTTAGATTTG
CTTCACTATTTCACTGTGACCTGTTACTCTGGTTATGGAGATGATGAAAAGGGCTTTTATACAGTCTATCGTA
ATGTTTTTGAAATGATTGCGAAGGAAGAACTAGAATCTGTGTTAGAAGAGGAGATGGAAGATTTCCCCAC
TTTTGGAGACTCCCAGAGCGACTATGATACGGTAGTCCATCCTTTCTATGCCTATTGGCAGAGTTTCTGCAC
TCAGAAGAGCTTTGCTTGGAAGGAAGAATATGATACACGACAAGCTTCAAACCGCTGGGAGAAACGAGC
CATGGAAAAAGAAAACAAAAAGATTCGCGACAAAGCAAGGAAGGAGAAGAATGAGCTTGTCCGTCAGC
TGGTTGCGTTCATCCGGAAAAGAGACAGGAGAGTGCAGGCGCATCGGAAGCTCGTGGAGGAACAGAAC
GCTGAGAAGGCGAGGAAGGCAGAGGAGATGAGGCGGCAGCAGAAGCTGAAGCAGGCAAAGCTGGCA
GAGCAGTACAGAGAGCAGAGCTGGATGACCGTGGCCGACCtggagaaggagctcaaggagatggaggcgcgctatg
agaaggagtttggagatggatcagatgaagaggagatggagGAATCTGAACTCAAAGATGGGCACGAAGGTAAAGACA
GTGATGAAGCTGAGGATGCTGAGCTGTACGATGACCTTTACTGCCCAGCATGTGACAAATCTTTCAAGACT
GAAAAGGCTATGAGGAATCATGAAAAATCAAAGAAGCATCGGGAAATGGTTGCTTTGTTAAAACAACAGT
TGGAGGAGGAGGAAGAAAATTTTTCAGGACCTCAAACTGATGAAAATCCATTGAATGCCAATTCTGAGGA
AGAAATAGAAGATGCACCAAAACAAAAGCTTTCTAAAAAGCAGAAGAAAAAGAAACAGAAACCAGCACA
GAATTATGATGATAATTTTAATGAAAATGGAACTGAAGAAGGAGTAAAGGTTGAATCAGAAGATACCAATT
TAAATCAAGACTGTGCCAAAGAACTAGAAGGTAGTCCCCAAGCAAATAGCAGTGTCACAGAGACTGTCCA
ACTCTGTGATGATCCAAAAAGTGAAGCTAAGAATGCTCCTAAACCCAAAGGAAAGAAAGCCAAAGATACG
AAAAAATCTGTCAAAGTACCTGCTGAACCACAGACAGTGAGTGATGTTCTTATCAGCTGTACAACCTGCCA
TAGTGAATTTCCATCCCGGAATAAACTTTTCGACCATCTGAAAGCCACAGGTCATGCAAGAGCACCTTCATC
ATGTTCTTTAGGCAGCATAACAAGTAGTCGAAGCAAGAAAGAGAAACGTAAAAACAGATAGAAATTCTGC
CAACACTTTTTCTTTTTGATTGTCTCTAGATTTTGAAACCAAAAACTGAACTGAAATCATCTAAAGAGTTAA
AATTTCGGTGATCTGCAATTTATTGCATTGTGGAAG

Supplementary File 2-7: Predicted truncate
d DNAJC21 protein
>Predicted_Truncated
MKCHYEALGVRRDASEEELKKAYRKLALKWHPDKNLDNAAEAAEQFKLIQAAKKKKKKKKKKKKKKKKKKKK
KKKNPSSI
