# Supplementary figures and images for: A naturally occurring canine model of syndromic congenital microphthalmia
Source: G3 (Bethesda). 2024 Apr 29;14(6):jkae067. doi: 10.1093/g3journal/jkae067 (PMC11152080; doi:10.1093/g3journal/jkae067)

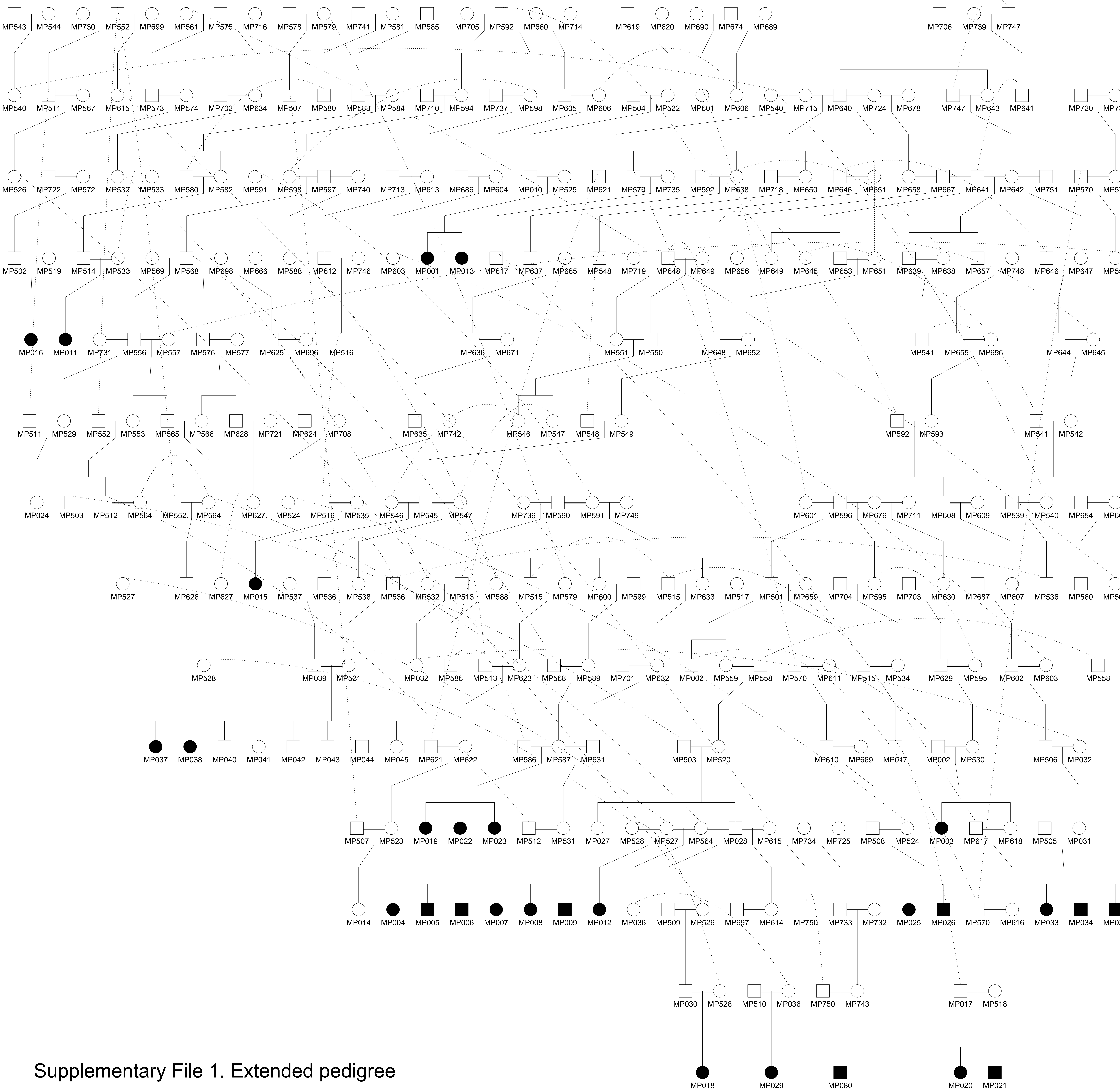

Supplementary File 1. Extended pedigree

Supplement: jkae067_Supplementary_Data [file jkae067_supplementary_data.zip › Supplementary_File_1_G3-2024-404875.pdf]
